# Supplementary material for: A cluster-randomized trial of client and provider-directed financial interventions to align incentives with appropriate case management in retail medicine outlets: Results of the TESTsmART Trial in western Kenya
Source: PLOS Glob Public Health. 2024 Feb 7;4(2):e0002451. doi: 10.1371/journal.pgph.0002451 (PMC10849268; doi:10.1371/journal.pgph.0002451)
Supplement: S1 Tables — (DOCX) [file pgph.0002451.s003.docx]

S1 Table: Trial outcomes definitions, numerator and denominators

| **Outcome** | **Client Sample** | **Client-Level Outcome** | **Summary Formula** | **Formula components** | **Denominator count** | **Numerator count** |
| --- | --- | --- | --- | --- | --- | --- |
| **Primary Outcome** |  |  |  |  |  |  |
| ACTs sold to malaria test-positive clients | All clients who purchased an ACT^^[[1]](#footnote-1)^^ | 1 = positive malaria test^^[[2]](#footnote-2)^^ and purchases ACT  0 = otherwise | $\frac{\# clients who purchased ACT and tested positive (n=902)}{\# clients who purchased ACT (N=3336)}$  Control = 313/1,039  CD = 250/1,004  CDPD = 339/1,293 | positive in outlet and bought AL or other ACT | 451+161 | 451+161 |
|  |  |  |  | negative in outlet and bought AL or other ACT | 334+59 | - |
|  |  |  |  | invalid test result and bought AL or other ACT | 25 | - |
|  |  |  |  | no test and bought AL or other ACT | 1487 +179 | - |
|  |  |  |  | positive test from outside outlet and bought AL or other ACT | 344+205 | 181+109^[[3]](#footnote-3)^ |
|  |  |  |  | negative test from outside outlet and bought AL or other ACT | 72+17 | - |
|  |  |  |  | unknown test from outside outlet and bought AL or other ACT | 2 | - |
|  |  |  |  |  | Total = 3336 | Total = 902 |
| **Secondary Outcomes** |  |  |  |  |  |  |
| Suspected malaria cases that receive a mRDT (testing uptake) | All suspected malaria cases^^[[4]](#footnote-4)^^ | 1 = tested with  mRDT^^[[5]](#footnote-5)^^  0 = otherwise | $\frac{\# suspected malaria cases tested with mRDT (n=2436 )}{\# suspected malaria cases (N=4995)}$  Control = 819/1,541  CD = 804/1,576  CDPD = 813/1,878 | Tested at outlet (mRDT) | 2436 | 2436 |
|  |  |  |  | No test and bought AL, other ACT, or other AM | 1487+179+204 | - |
|  |  |  |  | positive test elsewhere and bought AL, other ACT, or other AM | 344+205+44 | - |
|  |  |  |  | negative test elsewhere and bought AL, other ACT, or other AM | 72+17+4 | - |
|  |  |  |  | Unknown test result from elsewhere and bought AL, ACT, or other AM | 3 | - |
|  |  |  |  |  | Total = 4995 | Total = 2436 |
| Malaria tested clients whose treatment adhered to test results | All clients who were tested with mRDT^^[[6]](#footnote-6)^^ | 1 = positive malaria test and purchases ACT OR negative malaria test and did not purchase any AM  0 = otherwise | $\begin{matrix} \#clients that tested positive with mRDT and purchased ACT+ \\ \frac{\# clients that tested negative with mRDTand did not purchase AM (n=1744)}{\# clients tested with mRDT (N=2436)} \end{matrix}$  Control = 558/819  CD = 595/804  CDPD = 591/813 | positive in outlet and bought AL or other ACT | 451+161 | 451+161 |
|  |  |  |  | positive in outlet and did not buy AL or other ACT | 842-(451+161) | - |
|  |  |  |  | negative in outlet and bought AL, other ACT, or other AM | 334+59+29 | - |
|  |  |  |  | negative in outlet and did not buy AM | 1554-(334+59+29) | 1554-(334+59+29) |
|  |  |  |  | invalid/unknown test result at outlet | 40 | - |
|  |  |  |  |  | Total = 2436 | Total = 1744 |
| Suspected malaria cases that are managed appropriately | All suspected malaria cases | 1 = positive malaria test and purchases ACT OR negative malaria test and did not purchase any AM  0 = otherwise | $\begin{matrix} \#clients that tested positive with mRDT and purchased ACT+ \\ \frac{\# clients that tested negative with mRDT and did not purchase AM (n=1744)}{\# suspected malaria cases (N=4995)} \end{matrix}$  Control = 558/1,541  CD = 595/1,576  CDPD = 591/1,878 | positive in outlet and bought AL or other ACT | 451+161 | 451+161 |
|  |  |  |  | positive in outlet and did not buy AL or other ACT | 842-(451+161) | - |
|  |  |  |  | negative in outlet and bought AL, other ACT, or other AM | 334+59+29 | - |
|  |  |  |  | negative in outlet and did not buy AM | 1554-(334+59+29) | 1554-(334+59+29) |
|  |  |  |  | invalid/unknown test result at outlet | 40 | - |
|  |  |  |  | No test and bought AL, other ACT, or other AM | 1487+179+204 | - |
|  |  |  |  | positive test elsewhere and bought AL, other ACT, or other AM | 344+205+44 | - |
|  |  |  |  | negative test elsewhere and bought AL, other ACT, or other AM | 72+17+4 | - |
|  |  |  |  | Unknown test result from elsewhere and bought AL, ACT, or other AM | 3 | - |
|  |  |  |  |  | Total = 4995 | Total = 1744 |
| Untested clients taking ACT | All untested^^[[7]](#footnote-7)^^ clients | 1= purchased ACT  0 = otherwise | $\frac{\# untested clients who purchased ACT (n=1666)}{\# untested clients (N=2220)}$  Control = 523/676  CD = 542/746  CDPD = 601/798 | No test and bought AL other ACT | 1487+179 | 1487+179 |
|  |  |  |  | No test and did not buy AL or other ACT | 2220-(1487+179) | - |
|  |  |  |  |  | Total = 2220 | Total = 1666 |

S2 Table: Testing and test adherence by participant characteristics

|  | **Testing uptake at outlet** | **Adherence to mRDT results** | | |
| --- | --- | --- | --- | --- |
| **Characteristic** | **N = 5,695** | **Negative mRDT,**  **N = 1,554** | **Positive mRDT,**  **N = 842** | **Overall,**  **N = 2,396** |
| Adult client gender, n/N (%) |  |  |  |  |
| Male | 665/2,117 (31%) | 327/450 (73%) | 158/204 (77%) | 485/654 (74%) |
| Female | 846/1,828 (46%) | 456/576 (79%) | 182/255 (71%) | 638/831 (77%) |
| Adult client education level, n/N (%) |  |  |  |  |
| Primary or less | 500/1,347 (37%) | 249/318 (78%) | 125/171 (73%) | 374/489 (76%) |
| Beyond primary | 988/2,519 (39%) | 525/698 (75%) | 209/276 (76%) | 734/974 (75%) |
| Child client gender, n/N (%) |  |  |  |  |
| Male | 485/899 (54%) | 174/270 (64%) | 147/207 (71%) | 321/477 (67%) |
| Female | 440/851 (52%) | 175/258 (68%) | 125/176 (71%) | 300/434 (69%) |
| Respondent for child gender, n/N (%) |  |  |  |  |
| Male | 215/404 (53%) | 73/109 (67%) | 72/101 (71%) | 145/210 (69%) |
| Female | 707/1,341 (53%) | 275/418 (66%) | 198/280 (71%) | 473/698 (68%) |
| Respondent for child education level, n/N (%) |  |  |  |  |
| Primary or less | 316/606 (52%) | 108/162 (67%) | 89/147 (61%) | 197/309 (64%) |
| Beyond primary | 590/1,101 (54%) | 233/355 (66%) | 180/229 (79%) | 413/584 (71%) |
| Wealth quintile, n/N (%) |  |  |  |  |
| 0 to 20th | 406/1,078 (38%) | 183/243 (75%) | 105/156 (67%) | 288/399 (72%) |
| >20.0 to 40th | 444/1,073 (41%) | 213/287 (74%) | 107/150 (71%) | 320/437 (73%) |
| >40.0 to 60th | 495/1,080 (46%) | 238/329 (72%) | 123/161 (76%) | 361/490 (74%) |
| >60.0 to 80th | 501/1,135 (44%) | 239/319 (75%) | 129/175 (74%) | 368/494 (74%) |
| >80.0 | 461/1,004 (46%) | 204/297 (69%) | 119/157 (76%) | 323/454 (71%) |
| Client age, n/N (%) |  |  |  |  |
| Under 5 years | 388/793 (49%) | 163/218 (75%) | 120/163 (74%) | 283/381 (74%) |
| 5-17 years | 537/957 (56%) | 186/310 (60%) | 152/220 (69%) | 338/530 (64%) |
| 18+ years | 1,511/3,945 (38%) | 783/1,026 (76%) | 340/459 (74%) | 1,123/1,485 (76%) |
|  | | | | |

*S3a Table: Primary Outcome - ACTs sold to malaria test-positive clients*

*GEE analysis with KC Small Sample Variance Correction, N (minimally adjusted) = 3336, N (fully adjusted) = 3123*

|  | **Risk Ratio** | | **Risk Difference** | |
| --- | --- | --- | --- | --- |
| **Characteristic** | **Minimally Adjusted RR (95% CI)^1^** | **Fully Adjusted RR (95% CI)^1^** | **Minimally Adjusted RD (95% CI)^1^** | **Fully Adjusted RD (95% CI)^1^** |
| Arm, sample proportion n/N (%) |  |  |  |  |
| CDPD, 339/1,293 (26%) | — | — | — | — |
| CD, 250/1,004 (25%) | 0.92 (0.49 to 1.71) | 0.94 (0.53 to 1.66) | -0.05 (-0.22 to 0.11) | -0.05 (-0.20 to 0.09) |
| Control, 313/1,039 (30%) | 1.11 (0.61 to 2.02) | 1.15 (0.66 to 2.00) | 0.00 (-0.16 to 0.17) | 0.01 (-0.14 to 0.16) |
| period | 0.93 (0.82 to 1.06) | 0.94 (0.83 to 1.06) | -0.02 (-0.05 to 0.01) | -0.03 (-0.06 to 0.00) |
| period_quadratic | 1.01 (0.99 to 1.02) | 1.01 (0.99 to 1.02) | 0.00 (0.00 to 0.01) | 0.00 (0.00 to 0.01) |
| period_cubic | 1.00 (1.00 to 1.00) | 1.00 (1.00 to 1.00) | 0.00 (0.00 to 0.00) | 0.00 (0.00 to 0.00) |
| County |  |  |  |  |
| Bungoma | — | — | — | — |
| Transnzoia | 1.31 (0.82 to 2.10) | 1.24 (0.81 to 1.92) | 0.09 (-0.03 to 0.21) | 0.08 (-0.03 to 0.19) |
| Client gender |  |  |  |  |
| Male |  | — |  | — |
| Female |  | 1.21 (1.07 to 1.37) |  | 0.07 (0.03 to 0.10) |
| Client age |  |  |  |  |
| Under 5 years |  | — |  | — |
| 5-17 years |  | 0.93 (0.77 to 1.13) |  | -0.03 (-0.11 to 0.05) |
| 18+ years |  | 0.51 (0.44 to 0.59) |  | -0.22 (-0.27 to -0.16) |
| Education level |  |  |  |  |
| None |  | — |  | — |
| Primary |  | 1.09 (0.82 to 1.44) |  | 0.01 (-0.05 to 0.08) |
| Secondary |  | 1.01 (0.74 to 1.37) |  | -0.01 (-0.08 to 0.06) |
| College |  | 1.06 (0.77 to 1.47) |  | 0.02 (-0.06 to 0.09) |
| University |  | 1.21 (0.79 to 1.84) |  | 0.05 (-0.05 to 0.16) |
| Wealth index (quintile) |  |  |  |  |
| 0 to 20th |  | — |  | — |
| >20.0 to 40th |  | 1.08 (0.86 to 1.36) |  | 0.01 (-0.03 to 0.05) |
| >40.0 to 60th |  | 1.15 (0.96 to 1.38) |  | 0.03 (-0.01 to 0.07) |
| >60.0 to 80th |  | 1.14 (0.91 to 1.42) |  | 0.04 (-0.02 to 0.09) |
| >80.0 |  | 1.30 (0.95 to 1.78) |  | 0.06 (-0.03 to 0.16) |
| **^1^RR = Risk Ratio, RD = Risk Difference, CI = Confidence Interval** | | | | |

*S3b Table: Secondary Outcome 1 - Suspected malaria cases that receive a mRDT (testing uptake)*

*GEE analysis with KC Small Sample Variance Correction, N (minimally adjusted) = 4995, N (fully adjusted) = 4680*

|  | **Risk Ratio** | | **Risk Difference** | |
| --- | --- | --- | --- | --- |
| **Characteristic** | **Minimally Adjusted RR (95% CI)^1^** | **Fully Adjusted RR (95% CI)^1^** | **Minimally Adjusted RD (95% CI)^1^** | **Fully Adjusted RD (95% CI)^1^** |
| Arm, sample proportion n/N (%) |  |  |  |  |
| CDPD, 813/1,878 (43%) | — | — | — | — |
| CD, 804/1,576 (51%) | 1.15 (0.60 to 2.18) | 1.14 (0.60 to 2.17) | 0.04 (-0.14 to 0.21) | 0.07 (-0.09 to 0.22) |
| Control, 819/1,541 (53%) | 1.22 (0.64 to 2.33) | 1.20 (0.63 to 2.30) | 0.09 (-0.11 to 0.29) | 0.09 (-0.08 to 0.26) |
| period | 0.89 (0.83 to 0.95) | 0.89 (0.83 to 0.96) | -0.07 (-0.09 to -0.04) | -0.06 (-0.09 to -0.03) |
| period_quadratic | 1.02 (1.02 to 1.03) | 1.02 (1.01 to 1.03) | 0.01 (0.01 to 0.02) | 0.01 (0.01 to 0.01) |
| period_cubic | 1.00 (1.00 to 1.00) | 1.00 (1.00 to 1.00) | 0.00 (0.00 to 0.00) | 0.00 (0.00 to 0.00) |
| County |  |  |  |  |
| Bungoma | — | — | — | — |
| Transnzoia | 1.30 (0.77 to 2.20) | 1.25 (0.74 to 2.13) | 0.12 (-0.02 to 0.27) | 0.11 (-0.02 to 0.24) |
| Client gender |  |  |  |  |
| Male |  | — |  | — |
| Female |  | 1.28 (1.20 to 1.36) |  | 0.12 (0.09 to 0.15) |
| Client age |  |  |  |  |
| Under 5 years |  | — |  | — |
| 5-17 years |  | 1.02 (0.94 to 1.10) |  | 0.01 (-0.07 to 0.09) |
| 18+ years |  | 0.69 (0.59 to 0.82) |  | -0.19 (-0.28 to -0.10) |
| Education level |  |  |  |  |
| None |  | — |  | — |
| Primary |  | 0.91 (0.83 to 1.01) |  | -0.04 (-0.12 to 0.04) |
| Secondary |  | 0.92 (0.82 to 1.03) |  | -0.04 (-0.12 to 0.03) |
| College |  | 0.89 (0.78 to 1.02) |  | -0.06 (-0.14 to 0.02) |
| University |  | 0.82 (0.67 to 1.00) |  | -0.08 (-0.18 to 0.03) |
| Wealth index (quintile) |  |  |  |  |
| 0 to 20th |  | — |  | — |
| >20.0 to 40th |  | 1.06 (0.97 to 1.15) |  | 0.03 (-0.01 to 0.08) |
| >40.0 to 60th |  | 1.09 (0.94 to 1.25) |  | 0.05 (0.01 to 0.08) |
| >60.0 to 80th |  | 1.05 (0.88 to 1.25) |  | 0.03 (-0.02 to 0.08) |
| >80.0 |  | 1.08 (0.83 to 1.41) |  | 0.04 (-0.02 to 0.11) |
| **^1^RR = Risk Ratio, RD = Risk Difference, CI = Confidence Interval** | | | | |

*S3c Table: Secondary Outcome 2 - mRDT tested clients whose treatment adhered to test results*

*GEE analysis with KC Small Sample Variance Correction, N (minimally adjusted) = 2436, N (fully adjusted) = 2295*

|  | **Risk Ratio** | | **Risk Difference** | |
| --- | --- | --- | --- | --- |
| **Characteristic** | **Minimally Adjusted RR (95% CI)^1^** | **Fully Adjusted RR (95% CI)^1^** | **Minimally Adjusted RD (95% CI)^1^** | **Fully Adjusted RD (95% CI)^1^** |
| Arm, sample proportion n/N (%) |  |  |  |  |
| CDPD, 591/813 (73%) | — | — | — | — |
| CD, 595/804 (74%) | 0.99 (0.52 to 1.90) | 1.00 (0.52 to 1.93) | -0.01 (-0.13 to 0.11) | 0.00 (-0.12 to 0.11) |
| Control, 558/819 (68%) | 0.94 (0.46 to 1.90) | 0.94 (0.47 to 1.91) | -0.06 (-0.17 to 0.05) | -0.05 (-0.16 to 0.07) |
| period | 0.96 (0.91 to 1.02) | 0.97 (0.91 to 1.04) | -0.03 (-0.06 to 0.00) | -0.02 (-0.06 to 0.01) |
| period_quadratic | 1.01 (1.00 to 1.02) | 1.01 (1.00 to 1.01) | 0.00 (0.00 to 0.01) | 0.00 (0.00 to 0.01) |
| period_cubic | 1.00 (1.00 to 1.00) | 1.00 (1.00 to 1.00) | 0.00 (0.00 to 0.00) | 0.00 (0.00 to 0.00) |
| County |  |  |  |  |
| Bungoma | — | — | — | — |
| Transnzoia | 1.11 (0.64 to 1.93) | 1.10 (0.63 to 1.94) | 0.08 (-0.01 to 0.17) | 0.08 (-0.01 to 0.17) |
| Client gender |  |  |  |  |
| Male |  | — |  | — |
| Female |  | 1.03 (0.99 to 1.08) |  | 0.03 (-0.01 to 0.06) |
| Client age |  |  |  |  |
| Under 5 years |  | — |  | — |
| 5-17 years |  | 0.87 (0.73 to 1.04) |  | -0.09 (-0.16 to -0.02) |
| 18+ years |  | 1.01 (0.84 to 1.22) |  | 0.01 (-0.04 to 0.06) |
| Education level |  |  |  |  |
| None |  | — |  | — |
| Primary |  | 1.00 (0.85 to 1.18) |  | 0.01 (-0.09 to 0.10) |
| Secondary |  | 1.05 (0.90 to 1.22) |  | 0.04 (-0.07 to 0.15) |
| College |  | 1.03 (0.86 to 1.24) |  | 0.03 (-0.09 to 0.15) |
| University |  | 1.17 (0.95 to 1.44) |  | 0.13 (0.02 to 0.25) |
| Wealth index (quintile) |  |  |  |  |
| 0 to 20th |  | — |  | — |
| >20.0 to 40th |  | 1.00 (0.87 to 1.15) |  | 0.00 (-0.08 to 0.08) |
| >40.0 to 60th |  | 0.99 (0.82 to 1.19) |  | -0.01 (-0.07 to 0.05) |
| >60.0 to 80th |  | 1.00 (0.81 to 1.23) |  | 0.00 (-0.06 to 0.05) |
| >80.0 |  | 0.95 (0.69 to 1.31) |  | -0.04 (-0.11 to 0.04) |
| **^1^RR = Risk Ratio, RD = Risk Difference, CI = Confidence Interval** | | | | |

*S3d Table: Secondary Outcome 3 - Suspected malaria cases that are managed appropriately*

*GEE analysis with KC Small Sample Variance Correction, N (minimally adjusted) = 4995, N (fully adjusted) = 4680*

|  | **Risk Ratio** | | **Risk Difference** | |
| --- | --- | --- | --- | --- |
| **Characteristic** | **Minimally Adjusted RR (95% CI)^1^** | **Fully Adjusted RR (95% CI)^1^** | **Minimally Adjusted RD (95% CI)^1^** | **Fully Adjusted RD (95% CI)^1^** |
| Arm, sample proportion n/N (%) |  |  |  |  |
| CDPD, 591/1,878 (31%) | — | — | — | — |
| CD, 595/1,576 (38%) | 1.15 (0.58 to 2.29) | 1.15 (0.58 to 2.29) | 0.02 (-0.13 to 0.17) | 0.04 (-0.10 to 0.17) |
| Control, 558/1,541 (36%) | 1.14 (0.57 to 2.29) | 1.13 (0.56 to 2.27) | 0.03 (-0.12 to 0.17) | 0.03 (-0.10 to 0.16) |
| period | 0.86 (0.80 to 0.92) | 0.87 (0.81 to 0.94) | -0.06 (-0.08 to -0.04) | -0.06 (-0.08 to -0.04) |
| period_quadratic | 1.03 (1.02 to 1.04) | 1.03 (1.02 to 1.04) | 0.01 (0.01 to 0.01) | 0.01 (0.01 to 0.01) |
| period_cubic | 1.00 (1.00 to 1.00) | 1.00 (1.00 to 1.00) | 0.00 (0.00 to 0.00) | 0.00 (0.00 to 0.00) |
| County |  |  |  |  |
| Bungoma | — | — | — | — |
| Transnzoia | 1.44 (0.82 to 2.54) | 1.39 (0.78 to 2.45) | 0.12 (0.01 to 0.23) | 0.11 (0.00 to 0.21) |
| Client gender |  |  |  |  |
| Male |  | — |  | — |
| Female |  | 1.33 (1.25 to 1.42) |  | 0.10 (0.07 to 0.13) |
| Client age |  |  |  |  |
| Under 5 years |  | — |  | — |
| 5-17 years |  | 0.89 (0.81 to 0.98) |  | -0.05 (-0.12 to 0.01) |
| 18+ years |  | 0.71 (0.60 to 0.86) |  | -0.14 (-0.21 to -0.07) |
| Education level |  |  |  |  |
| None |  | — |  | — |
| Primary |  | 0.92 (0.83 to 1.03) |  | -0.03 (-0.11 to 0.05) |
| Secondary |  | 0.97 (0.85 to 1.11) |  | -0.02 (-0.11 to 0.07) |
| College |  | 0.92 (0.79 to 1.08) |  | -0.03 (-0.12 to 0.06) |
| University |  | 0.96 (0.77 to 1.19) |  | 0.00 (-0.10 to 0.10) |
| Wealth index (quintile) |  |  |  |  |
| 0 to 20th |  | — |  | — |
| >20.0 to 40th |  | 1.05 (0.95 to 1.17) |  | 0.02 (-0.02 to 0.07) |
| >40.0 to 60th |  | 1.08 (0.92 to 1.26) |  | 0.02 (-0.01 to 0.06) |
| >60.0 to 80th |  | 1.04 (0.86 to 1.25) |  | 0.01 (-0.03 to 0.06) |
| >80.0 |  | 1.02 (0.77 to 1.36) |  | 0.01 (-0.04 to 0.06) |
| **^1^RR = Risk Ratio, RD = Risk Difference, CI = Confidence Interval** | | | | |

*S3e Table: Secondary Outcome 4 - Untested clients taking ACT*

*GEE analysis with KC Small Sample Variance Correction, N (minimally adjusted) = 2220, N (fully adjusted) = 2071*

|  | **Risk Ratio** | | **Risk Difference** | |
| --- | --- | --- | --- | --- |
| **Characteristic** | **Minimally Adjusted RR (95% CI)^1^** | **Fully Adjusted RR (95% CI)^1^** | **Minimally Adjusted RD (95% CI)^1^** | **Fully Adjusted RD (95% CI)^1^** |
| Arm, sample proportion n/N (%) |  |  |  |  |
| CDPD, 601/798 (75%) | — | — | — | — |
| CD, 542/746 (73%) | 0.96 (0.51 to 1.82) | 0.96 (0.50 to 1.82) | -0.03 (-0.09 to 0.03) | -0.03 (-0.08 to 0.02) |
| Control, 523/676 (77%) | 1.03 (0.55 to 1.91) | 1.01 (0.54 to 1.88) | 0.02 (-0.04 to 0.08) | 0.01 (-0.06 to 0.07) |
| period | 0.99 (0.91 to 1.08) | 0.98 (0.89 to 1.07) | -0.01 (-0.04 to 0.02) | -0.02 (-0.05 to 0.01) |
| period_quadratic | 1.00 (0.99 to 1.01) | 1.00 (0.99 to 1.01) | 0.00 (0.00 to 0.00) | 0.00 (0.00 to 0.01) |
| period_cubic | 1.00 (1.00 to 1.00) | 1.00 (1.00 to 1.00) | 0.00 (0.00 to 0.00) | 0.00 (0.00 to 0.00) |
| County |  |  |  |  |
| Bungoma | — | — | — | — |
| Transnzoia | 0.99 (0.60 to 1.64) | 1.00 (0.60 to 1.66) | -0.01 (-0.05 to 0.04) | 0.00 (-0.05 to 0.04) |
| Client gender |  |  |  |  |
| Male |  | — |  | — |
| Female |  | 0.94 (0.86 to 1.02) |  | -0.05 (-0.10 to 0.00) |
| Client age |  |  |  |  |
| Under 5 years |  | — |  | — |
| 5-17 years |  | 1.18 (1.00 to 1.41) |  | 0.12 (0.03 to 0.20) |
| 18+ years |  | 1.15 (1.00 to 1.33) |  | 0.10 (0.03 to 0.17) |
| Education level |  |  |  |  |
| None |  | — |  | — |
| Primary |  | 0.99 (0.85 to 1.15) |  | -0.01 (-0.11 to 0.09) |
| Secondary |  | 1.05 (0.86 to 1.28) |  | 0.03 (-0.07 to 0.14) |
| College |  | 1.07 (0.86 to 1.33) |  | 0.05 (-0.06 to 0.16) |
| University |  | 1.07 (0.85 to 1.34) |  | 0.06 (-0.08 to 0.20) |
| Wealth index (quintile) |  |  |  |  |
| 0 to 20th |  | — |  | — |
| >20.0 to 40th |  | 1.01 (0.91 to 1.12) |  | 0.01 (-0.05 to 0.07) |
| >40.0 to 60th |  | 1.08 (0.95 to 1.22) |  | 0.06 (0.01 to 0.10) |
| >60.0 to 80th |  | 1.06 (0.91 to 1.24) |  | 0.05 (-0.01 to 0.10) |
| >80.0 |  | 0.99 (0.80 to 1.23) |  | -0.01 (-0.06 to 0.04) |
| **^1^RR = Risk Ratio, RD = Risk Difference, CI = Confidence Interval** | | | | |

Table S4: Injections reported by test status and arm

|  | **Test status** | | | **Study Arm** | | | |
| --- | --- | --- | --- | --- | --- | --- | --- |
| **Characteristic** | **No test, N = 2,911^1^** | **Positive test (any source), N = 1,230^1^** | **Negative test (shop), N = 1,554^1^** | **Control, N = 1,692^1^** | **CD, N = 1,789^1^** | **CDPD, N = 2,214^1^** | **Overall^1^** |
| Any injection reported | 105 (3.6%) | 272 (22%) | 87 (5.6%) | 186 (11%) | 130 (7.3%) | 148 (6.7%) | 464 (8.1%) |
| Injection type |  |  |  |  |  |  |  |
| Identified as artemisinin injection  *Artemisinin injection plus oral ACT (AL or DP)* | 29 (28%)  *8* | 46 (17%)  *16* | 13 (15%)  *4* | 32 (17%)  *11* | 17 (13%)  *4* | 40 (27%)  *13* | 89 (19%)  *28* |
| Identified as 'malaria injection' or 'antimalarial injection' or quinine injection  *'Malaria' injection plus oral ACT (AL or DP)* | 13 (12%)    *2* | 64 (24%)  *18* | 5 (5.7%)  *2* | 30 (16%)  *5* | 30 (23%)  *12* | 22 (15%)  *5* | 82 (18%)  *22* |
| Painkiller or antibiotic injection | 13 (12%) | 6 (2.2%) | 12 (14%) | 9 (4.8%) | 8 (6.2%) | 14 (9.5%) | 31 (6.7%) |
| Unspecified injection  *Unspecified injection plus oral ACT (AL or DP)* | 54 (51%)  *20* | 156 (57%)  *77* | 59 (68%)  *4* | 117 (63%)  *40* | 76 (58%)  *28* | 76 (51%)  *33* | 269 (58%)  *101* |
| **^1^n (%)** | | | | | | | |

1. For primary analysis, ACTs will include both study approved AL and other ACTs. [↑](#footnote-ref-1)
2. mRDT+ based on RDT conducted at retail outlet or positive by test (either RDT or microscopy) conducted outside of the retail outlet provided that documentation is provided [↑](#footnote-ref-2)
3. The values 181 and 109 represent only participants who showed documentation of a positive malaria test from elsewhere and purchased AL or ACT medications, respectively, whereas the “Took AL” and “Took ACT (not AL)” rows under “Had positive test from elsewhere” in Table 2 also include participants who reported a positive test from elsewhere but did not present documentation of the test. [↑](#footnote-ref-3)
4. A “suspected malaria case” is any client who was tested with an mRDT or was untested but purchased any antimalarial (AM) [↑](#footnote-ref-4)
5. mRDT at the outlet [↑](#footnote-ref-5)
6. mRDT at the outlet [↑](#footnote-ref-6)
7. Neither tested at outlet nor reported results from test elsewhere [↑](#footnote-ref-7)
