# Supplementary material for: A cluster-randomized trial of client and provider-directed financial interventions to align incentives with appropriate case management in retail medicine outlets: Results of the TESTsmART Trial in western Kenya
Source: PLOS Glob Public Health. 2024 Feb 7;4(2):e0002451. doi: 10.1371/journal.pgph.0002451 (PMC10849268; doi:10.1371/journal.pgph.0002451)
Supplement: S1 Text — (PDF) [file pgph.0002451.s004.pdf]

Statistical Analysis Plan (SAP)

**Title**	A randomized experiment of malaria diagnostic testing and conditional subsidies to target ACTs in the retail sector: TESTsmART trial AIM 2
**CRU/Department/Division/Centre**	Duke Global Health Institute<br>Clinton Health Access Initiative (CHAI)<br>Moi University
**IRB Number**	TESTsmART Pro00104256
**Investigators:**	
**Lead Investigator**	Wendy Prudhomme O'Meara
**Mentors**	
**Co-authors (if known)**	Theodoor Visser<br>Jeremiah Laktabai
**Biostatistician(s)**	Ryan Simmons<br>Yunji Zhou<br>David Arthur<br>John Gallis
**Supervising Biostatistician**	Elizabeth Louise Turner
**Original Creation Date**	01/11/2021
**Version Date**	10/28/2022
**Project Folder Location**	"~\Box\PROJECT_DGHI_TESTsmART_wpo "
**Project Goal(s)**	Manuscript

Investigator Agreement

- ☐ All statistical analyses included in an abstract or manuscript should reflect the work of the biostatistician(s) listed on this SAP. No changes or additional analyses should be made to the results or findings without discussing with the project biostatistician(s).
- ☐ All biostatisticians on this SAP should be given sufficient time to review the full presentation, abstract, manuscript, or grant and be included as co-authors on any abstract or manuscript resulting from the analyses.

	☐ Publications resulting from this SAP are supported in part by the Duke CTSA and must cite grant number UL1TR002553 and be submitted to PubMed Central.<br>☐I have reviewed the SAP and understand that any changes must be documented.<br><br>*Acknowledged by:* Wendy O'Meara<br>*Date:* December 1, 2022<br><br>*Acknowledged by:* Theodoor Visser<br>*Date:* December 1, 2022
**Activity Log**	2021-02-02: Discussed selection bias and intervention characteristics<br>2022-06-14-2022-06-24: John, David, Yunji and Liz made additional comments/edits to all sections. In particular, to sample size, outcome definitions and expanded on the methods section to include the paired GEE/MAEE approach<br>2022-06-24: John reviewed comments and made additional edits.<br>2022-06-29: David reviewed comments and made additional edits.<br>2022-08-12: David added comments and added variable names to table shell B7(Missingness by Treatment Group)<br>2022-08-31: John reviewing SAP and making formatting edits<br>2022-09-19: David inserted the updated outcomes definitions section, did some formatting, and marked some comments as resolved.<br>2022-10-05: Liz expanded on the description of analyses and reviewed entire document.<br>2022-10-10: John added comments from Wendy to this document.<br>2022-10-17: John made edits and added comments during in-person meeting.<br>2022-10-21: David edited tables based on 10-17 discussion.<br>2022-10-26: David and John made small edits in response to comments.<br>2022-10-28: Liz minor edits<br>2022-11-15: David incorporates comments from 11-9 team meeting<br>2022-11-16: John updates in relation to Liz's comments on adjusting for time in main model

	2022-11-16: David updates in relation to John's comments<br>2022-11-17: Liz updates<br>2022-11-17: David updates<br>2022-11-18: David separates into separate document for Kenya<br>2022-11-28: David revises some variable names in tables to match code<br>2022-12-01: SAP finalized and approved by study PIs Wendy O'Meara and Theodoor Visser

1 Study Overview

In response to the high cost of artemisinin-combination therapy (ACT), publicly-funded, retail-sector ACT subsidies were adopted in many malaria-endemic countries. Declining prices of ACTs create a trade-off between access and targeting; lower prices improve uptake of effective therapies by those with malaria but also increase inappropriate use by those without malaria. Curbing inappropriate use and targeting ACTs to malaria cases requires parasitological diagnosis which is virtually absent in the retail sector. It is estimated that at least two thirds of ACTs purchased over-the-counter are consumed by individuals without malaria. Inappropriate use leads to wastage of public funds and prime conditions for the spread of drug resistant parasites, which could dramatically increase global mortality from malaria. Targeting subsidized ACTs to individuals with parasitologically-confirmed malaria would significantly contribute to the sustainability and cost-effectiveness of retail subsidies as well as safeguard the future efficacy of these key drugs. We propose an innovative conditional subsidy approach that links the ACT subsidy to the results of a malaria rapid diagnostic test (mRDT), allowing the subsidy to be targeted only to parasitologically-confirmed malaria cases. This targeted subsidy makes information from a test more valuable and could drive appropriate consumption while reducing costs.

In order to align both the provider and customer incentives towards testing and targeting, the provider must be willing to perform the test and sell the appropriate medicine and, at the same time, the customer must be motivated to purchase the test and adhere to the results. We hypothesize that offering ACT subsidies for the client (conditional on a positive test), will have a modest impact on uptake of testing and targeting of ACTs in the retail sector. We further hypothesize that when combined with a provider-incentive to offer malaria testing, they will have a synergistic effect on mRDT testing and ACT targeting. We will test the combination of subsidies for ACTs and mRDTs (selected from the study conducted as part of Aim 1 of the TESTsmART study) paired with a provider-directed testing incentive to evaluate their impact on the proportion of ACTs sold to individuals with parasitologically-confirmed malaria among those seeking care in the retail sector.

This study comprises a cluster randomized controlled trial focused on malaria diagnostic testing and conditional ACT subsidies with the goal to evaluate provider directed- and client-directed interventions. The study site is a rural region around Webuye in western Kenya. Clusters are 39 participating retail outlets. The diagnosis and treatment choices made during each transaction will be captured using a mobile phone app. Study outcomes will be collected through exit interviews with clients, who sought care for febrile illness, at each of the enrolled retail outlets. This study is linked to a similar cluster randomized

controlled trial in the contrasting urban study site of Lagos, Nigeria, where there are 48 participating retail outlets (SAP for Nigeria is contained in a separate document).

1.1 Study Aim

The aim of this second phase of the TESTsmART study is to evaluate the degree to which incentives, directed at the client or both the client and provider, affect the purchasing behavior of suspected malaria cases seeking treatment in the retail sector. In particular, we focus on clients' willingness to undergo diagnostic testing and to purchase appropriate treatments.

1.2 Interventions

The four intervention arms considered for evaluation are as follows:

1. Control intervention: mRDTs are made available at wholesale price to the retail outlet (25 Kenyan Shillings (KES) per test), and outlet owner/attendant is trained to use the mobile reporting app. mRDTs are offered to clients at a pre-determined price (KES 40 per test).
2. Provider-directed (PD) intervention: in addition to the interventions implemented in the control outlets, the retail outlet owner receives a small incentive (10 KES) to perform the mRDT (approximately USD $0.10 for each mRDT they conduct and report using the mobile app).
3. Client-directed (CD) intervention: in addition to the interventions implemented in the control outlets, clients visiting outlets in this arm receive a free ACT (cost equivalent to 150 Kenyan Shillings (KES) for adults and 60 KES for children) if they purchase an mRDT and receive a positive test result (conditional subsidy).
4. Combined (PD+CD) intervention: in addition to the interventions implemented in the control outlets, the retail outlet owner receives a small incentive (10 KES) to perform the mRDT (approximately USD $0.10 for each mRDT they conduct and report using the mobile app) and clients visiting outlets in this arm receive a free ACT (cost equivalent to 150 Kenyan Shillings (KES) for adults and 60 KES for children) if they purchase an mRDT and receive a positive test result (conditional subsidy), i.e., this intervention is a combination of the PD and CD interventions that are described in 2 and 3 above.

Although we had hoped that the trial could be a four-arm trial, in reality, it was not possible (see more information in Section 2.5 Randomization Procedure). Instead, three interventions will be compared: control, client-directed (CD), and combined (CD+PD). See the summary in **Table 1**, and further details in Sample Size Justification below.

Table 1. Intervention arms

Intervention Arm	Kenya
Control	x
Provider-directed (PD)	-
Client-directed (CD)	x
Combined (CD+PD)	x

1.3 Study Hypotheses

1.3.1 Primary

Two pairs of primary hypotheses are defined on the primary outcome, which is the proportion of ACTs that are sold to malaria test-positive clients.

H_{01}: The proportion of ACTs that are sold to malaria test-positive clients will be the same in the PD+CD arm compared to the control arm.
H_{A1}: The proportion of ACTs that are sold to malaria test-positive clients will be different in the PD+CD arm compared to the control arm.

H_{02}: The proportion of ACTs that are sold to malaria test-positive clients will be the same in the PD+CD arm compared to the CD arm.
H_{A2}: The proportion of ACTs that are sold to malaria test-positive clients will be different in the PD+CD arm compared to the CD arm.

We note that we did not power on the comparison of the CD arm to control arm and will examine this comparison as a secondary comparison. Details of the primary outcome can be found in Section 3. Details of the analytical methods can be found in Section 4.

1.3.2 Secondary

The major secondary outcome is the proportion of suspected malaria cases that are tested at the outlet, where we define a suspected malaria case as any client who was tested with an mRDT at the outlet or who was untested but purchased any antimalarial at the outlet. This outcome will allow us to determine whether the conditional subsidy can drive demand for testing at the outlet.

Other secondary outcomes include:

- Adherence to mRDT results (the proportion of clients who properly adhered to their mRDT result out of all clients receiving an mRDT)
- Appropriate case management (the proportion of clients who properly followed their mRDT result, with respect to follow-on treatment options, out of all suspected malaria cases).
- The proportion of untested clients purchasing ACTs, where untested clients are those who did not receive an mRDT at the outlet, and did not report having been tested elsewhere.

We defined adherence to the mRDT result as purchasing an ACT if they tested positive and not purchasing any antimalarial (AM) if they tested negative, and we define a client as a suspected malaria cases if they were tested with an mRDT or they were untested but purchased any AM.

The secondary outcomes will be evaluated using the same modelling approach as the primary analyses (see Section 4).

2 Study Population

Among those private registered retail outlets that expressed interest in participating in the study, eligible private outlets were identified according to set criteria:

2.1 Inclusion Criteria for Outlets

- Routinely stock and sell ACTs

- Willing to acquire mRDTs and use in diagnosing malaria for patients
- Willing to use a phone/app to collect/report data and receive incentives to conduct mRDTs
- Willing to allow a data collector to conduct patient exit interviews for several days each month at the outlet
- License/registration up to date

2.2 Exclusion Criteria for Outlets

- Having challenges with network connectivity at the outlet
- Participating in other NGO projects
- Having any agreements with drug/diagnostic marketers

Interviews will be conducted with clients departing from participating retail outlets (study clusters) on random days of the week. All clients exiting the outlet that day are eligible to be screened. Field researchers are instructed to make no pre-judgements about clients but rather approach the next available client exiting the outlet. Interviewees must meet specific eligibility criteria to proceed with the exit interview (see below). 170 clients per outlet are expected to enrol in the study over the course of the 15-month intervention period, noting that we will be well powered with as low as a median of 79 clients per outlet (see Power Justification). The study team will conduct a verbal consent process for participants who meet the inclusion/exclusion criteria, prior to participation in the exit interview outside the outlets.

2.3 Inclusion Criteria for Participants

- Participants with fever or history of fever in the last 48 hours or malaria-like illness
- Individual with malaria-like illness must be present at recruitment
- Older than one year of age

2.4 Exclusion Criteria for Participants

- Any individual with signs of severe illness requiring immediate referral
- Individuals who have taken an antimalarial in the last seven days, including for the current illness
- Be <18 years of age without a parent or legal guardian present
- Unable to consent

We note that there was initially debate as to whether to specify an additional exclusion criterion of someone who had already conducted a test elsewhere prior to visiting the retail outlet. Given that we may be interested in the behavior of such clients, this criterion was not added. Instead, analyses of some outcomes will exclude these individuals (see Section 3 below for a description of outcomes).

2.5 Randomization Procedure

Following enrolment, retail outlets were randomized to arms by the study statistician. We started with a list of 40 participating outlets. A uniform random number between 0 and 1 was generated for all 40 outlets, then sorted and split into 3 groups using the quantiles of the distribution stratified by county. Those groups were labelled A, B, C and those labels randomized so that they were allocated to one of the three trial arms. Given 40 outlets cannot be equally allocated to 3 arms, the extra outlet was assigned to the control arm. In addition, to avoid potential contamination of treatment effects, randomization was constrained such that any outlets in close proximity (<0.5km) were assigned to the same arm. (See details in Kenya randomization plan: "Box\PROJECT_DGHI_TESTsmART_wpo\TESTsmART_Aim 2 Prep\TESTsmART_Aim 2_Trial Design\Kenya Randomization\TESTsmART Aim 2 Kenya Randomization

20210421.docx"). We note that the eventual number of outlets was 39 due to the removal of outlets that relocated outside of the study area, and the current SAP focuses on analysis of data from the 39 outlets.

2.6 Power Justification

The primary comparison of interest is the effect on our primary outcome of offering a combination of provider-directed (PD) and client-directed (CD) interventions relative to the control arm (i.e. PD+CD arm vs. control arm). Two secondary comparisons were also of interest, namely CD vs. PD+CD arms and PD vs. PD+CD arms in order to evaluate whether the combination of provider-directed interventions with client-directed interventions has a larger effect than the client-directed intervention alone or than the provider-directed intervention alone. We powered the study to analyze significant changes in the aforementioned comparisons (see Table 2 below). We expect that each of the individual interventions (i.e. CD alone and PD alone) would each have a positive effect compared to the control arm, and that the largest effect would come from combining the two interventions (PD+CD).

We started with power calculations based on a four-arm design based on our original assumptions before we collected any pre-randomization pilot data. Later, a series of updated power calculations showed that it would not be viable to conduct a four-arm trial due to the limited power anticipated based on key assumptions and the constraint of the expected number of outlets (40). We note that this assessment was made using pre-randomization pilot data.

All power calculations were based on pairwise comparison of two proportions using the formula from Moulton and Hayes for comparing two proportions under a cluster-randomized trial design (1). We estimated the intra-class correlation coefficients (ICCs) for the primary outcome to be 0.009. We first determined the minimum sample sizes required for 90% power to detect the original hypothesized effect sizes for each of the three main comparisons of interest for a four-arm trial and chose the largest sample size per shop as the target sample size for all shops. To ensure overall two-tailed Type I error (alpha) control at 0.05 under the four-arm design, the conservative Bonferroni correction was used to fix the alpha level for each of the three comparisons at 0.05/3 = 0.0167 (2).

Pilot pre-randomization app data indicated slightly lower test positivity than expected; calculations based on this pilot data indicated we might only reach 59.5% power for the comparison of the combined PD+CD arm and the CD arm if we were to conduct a four-arm trial. By adjusting to a three-arm trial before randomization and increasing the number of clusters per arm (from 10 to 13), we expect to achieve 80.2% power for this comparison (**Table 2**). We decided to keep the CD arm, rather than PD arm, since private outlets are permitted to stock and sell ACTs but are not (yet) allowed to conduct mRDTs outside of research settings.

Table 2: Estimated power for primary outcome based on assumptions inspired by data collected via the app during initial three-month pilot data collection period before randomization

**Primary Outcome Comparison**	**3-arm design** With a total of 40 clusters, cluster size=170 (alpha=0.05/2 = 0.025)	
	Expected Effect Size	Power
Combined Interventions (PD+CD) vs. Control	23% (PD+CD) – 7% (Control) = **16 percentage points**	99.1%

Combined Interventions (PD+CD) vs. Provider-Directed Intervention (PD)	NA	NA
Combined Interventions (PD+CD) vs. Client-Directed Intervention (CD)	23% (PD+CD) – 12% (CD) = **11 percentage points**	80.2%
**Note**: Expected effect size, change in percentage of ACTs taken by clients with a positive test. Since our outcome is a composite measure of testing rates and adherence to the test result, our sample size calculations accounted for the fact that not everyone who we interview will have taken an ACT. Power was calculated based on 13 clusters per arm. In practice an additional cluster was expected to be available in the control arm (i.e. 40 clusters in total).		

We assessed the power using the aggregated exit interview data collected in the initial ten months. We used an updated ICC of 0.012, a lower cluster size of 79, a CV of cluster sizes of 0.47, and the updated key assumptions based on the aggregate exit interview data (details in "Box\TESTsmART_Duke Statistics Team\Collaboration Folder\Results\Aim 2 Assumption\TESTsmART Aim 2 (Kenya) Power Re-Calculation 20211109.pptx"). We found that we are still well powered even with a lower cluster size (**Table 4**). Therefore, further design change is not necessary.

Table 4: Estimated power for primary outcome based on assumptions updated with aggregated exit interview data from first ten months of implementation of the three-arm trial

	Power based on three-arm design	
Key Assumptions	13 clusters per arm (alpha=0.05/2=0.025);<br>m = 79; ICC = 0.012; CV = 0.47	
Primary Outcome Comparison	Expected Effect Size	Power
Combined Interventions (PD+CD) vs. Control Arm	30% (PD+CD) – 8% (Control) = **22 percentage points**	99.8%
Combined Interventions (PD+CD) vs. Client Directed Intervention (CD)	30% (PD+CD) – 14.6% (CD) = **15.4 percentage points**	86.0%

Notes: m, expected median cluster size by the end of the study; ICC, intraclass correlation coefficient; CV, coefficient of variation of cluster size.

Our sample originally included 40 retail outlets with 14 outlets assigned to the control arm and 13 outlets assigned to each of CD and PD+CD. In practice, one shop dropped out, resulting in 39 shops in total, with 13 in each arm. Within each of these outlets, we originally planned to have 170 exit interviews based on calculations performed pre-randomization. In practice, additional explorations of power based on aggregate data collected during the initial 10 months show that we expect to be well-powered with a median of 79/shop resulting in a total sample size of 3081 (79 X 39), recognizing that we may be able to recruit more individuals over the follow-up period.

2.7 Data Acquisition

Study design	Two/Three-arm cluster randomized controlled trial
Data source/how the data were collected	Data for participant exit interviews will be collected electronically via tablet. The data will be encrypted and password protected. Tablets are locked in a secure cabinet nightly and data are removed several times per week. The primary tool for developing the data collection forms will be REDCap hosted at Duke University.
Contact information for team member responsible for data collection/acquisition	Emmah Kimachas (kimachasnr@gmail.com)
Date or version (if downloaded, provide date)	
Data transfer method and date	Box
Where dataset is stored	"Box\TESTsmART_Duke Statistics Team\Data Files"

3 Outcomes, Exposures, and Additional Variables of Interest

All outcomes of interest are measured at the individual level and will be aggregated by each of the three trial arms. Importantly, not all outcomes will be defined on all individuals because some are "conditional" outcomes e.g. conditional on having taken a test. As a consequence, although the study design is a randomized experiment, we recognize that the analysis of some outcomes may be more subject to potential confounding or selection bias than that of outcomes that are defined for all individuals.

We further note that all outcome data is based on self-reported data collected from clients by research assistants at exit interviews. Therefore, for all outcomes, we assume that the client has complete, correct information about their interactions with personnel in the outlet and that the client correctly reports it. We note that, if the shop personnel provide incorrect information to the client, we would have limited ability to detect this for a given vendor-client interaction. As context and as an example of how incorrect information may be conveyed, a mystery shopper activity uncovered a situation whereby an mRDT was performed, the shopkeeper took the test behind the scenes, returned later with a positive test but the mystery shopper then tested negative on a second mRDT outside of the shop. This is very likely an example of the shopkeeper knowingly providing incorrect information.

Overall, we anticipate two kinds of potential reporting errors: (1) the shopkeeper provides incorrect information to the client, who then correctly reports that information, and, (2) the client misremembers (or misunderstands) or has incomplete information from the interaction with outlet personnel. We anticipate that both scenarios have a low probability of occurring. Our main concern would be that the misreporting is differential by study arm, so a question arises as to whether we could find ourselves in a

situation whereby we are not able to compare data of the same validity between arms. Again, we will struggle to determine whether such a situation arises in practice and therefore recognize it as a potential limitation.

3.1 Primary Outcome

Outcome	Description	Formula to calculate outcome	Variables and Source	Specifications
ACT consumption by parasitologically confirmed malaria cases	Proportion of ACTs[a] that are sold to malaria test-positive[b] clients.<br><br>Note that: This primary outcome definition of "ACTs" is any ACT (i.e. the three study-approved AL brands and also any other ACT). Additionally, we accept positive results from both the outlet and elsewhere with documentation as positive (i.e., as long as documentation is provided to the research assistant conducting the exit interview; that is, "test result from elsewhere = 2").	**Numerator:** # of clients who purchased ACT (ACT=1) and tested positive (test_results=2 OR test_else_res=1)<br><br>**Denominator:** # of clients who purchased ACT (ACT=1)<br><br>$\frac{\text{\#clients who purchased ACT and tested positive}}{\text{\#clients who purchased ACT}}$	Raw Items below	1 = positive malaria test[b] and purchases ACT<br><br>0 = otherwise
	As a sensitivity analysis, we will restrict ACT to only AL.<br><br>Note: given the difficulty in separating out AL types, and the fact that we started subsidizing all ALs a few months after the start of the trial (July 2021), we will consider all AL (i.e., type_medXX == 1, see below) for sensitivity analysis.	**Numerator:** # of clients who purchased AL (AL=1) and tested positive (test_results=2 OR test_else_res=1)<br><br>**Denominator:** # of clients who purchased AL (AL=1)<br><br>$\frac{\text{\#clients who purchased AL and tested positive}}{\text{\#clients who purchased AL}}$		

	Two other sensitivity analyses will analyze data collected only after a certain time point.<br><br>First, data up to the end of July 2021 will be excluded as this was prior to expansion of the list of which AL brands were subsidized.<br><br>Second, data up to the end of September 2021 will be excluded as this was prior to additional CHW outreach.	**Numerator:** # of clients who purchased ACT (ACT=1) and tested positive (test_results=2 OR test_else_res=2), after July 31, 2021 (post_expansion=1)<br><br>**Denominator:** # of clients who purchased ACT (ACT=1), after July 31, 2021 (post_expansion=1)<br><br>$\frac{\text{clients who purchased ACT and tested positive after July 31, 2021}}{\text{clients who purchased ACT after July 31, 2021}}$		
		**Numerator**: # of clients who purchased ACT (ACT=1) and tested positive (test_results=2 OR test_else_res=1), after September 30, 2021 (post_CHW=1)<br><br>**Denominator**: # of clients who purchased ACT (ACT=1), after September 30, 2021 (post_CHW=1)<br><br>$\frac{\text{clients who purchased ACT and tested positive after September 30, 2021}}{\text{clients who purchased ACT after September 30, 2021}}$		

Raw items used to calculate the primary outcome:

**Item**	**Description**	**Variables and Source**	**Specifications**
Test result from the outlet	mRDT results from outlet (self-report)	test_results	1 = Negative<br>2 = Positive<br>3 = Invalid

Test result from elsewhere	Malaria diagnostic test result before coming to the outlet (we only accept those with documentation observed)	test_else_res	1 = Negative<br>2 = Positive - report observed
Treatment purchasing behavior (entered by exit interviewer, which is considered unverified; this variable will **not** be used for analysis, but listed here for our reference)	Which medicine(s) did you obtain from the outlet today to treat your/your child's illness?<br><br>Note that type_medXX == 1 includes both study-approved AL and non-study AL.<br><br>type_medXX == 2 includes all other non-study ACTs.<br><br>**IMPORTANT:** There could be potential errors in this variable (purchase) entered by exit interviewers. Therefore, we verified the medication list with the help from field teams. Verified variables are listed below.	type_medXX<br><br>(type_med1, type_med2, …, type_med10)	1 = AL (Lonart/CoArtem/Artefan/Lumartem/Amatem)<br>2 = Other ACT (DHAP, DP, Duocotexin, P-alaxin)<br>3 = Other antimalarial (Artesunate/artemether, Quinine, Chloroquine, SP/Fansidar)<br>4 = Antibiotic (Amoxyl, Septrin, Metronidazole/Flagyl, Ampicillin)<br>5 = Painkiller/fever medicine (Panadol/Brufen/Hedex/Action/Maramoja)<br>6 = Cough medicine or decongestant<br>555 = Other<br>0 = None<br>999 = Don't know
Purchased AL (based on verified medication list)	Whether clients purchased any kind of AL.<br><br>In the sensitivity analysis, we include all AL because of the difficulty of separating out our original list of study-approved AL (i.e. Lonart, Coartem, and Lumartem) from the more extensive list of all AL (i.e. identified by type_medXX ==1). Moreover, the fact that we started subsidizing all ALs a few months after the start of the trial (July 2021) would also render it challenging to focus solely on the original list of study-approved AL. Therefore, we will consider all AL (i.e., AL==1) for this analysis.	AL (a new variable based on type_medXX == 1, but with verification from field teams)	1 = yes<br>0 = no
Other, non-AL ACT (based on	Whether clients purchased non-AL ACT.	other_ACT (a new variable based on type_medXX == 2,	1 = yes<br>0 = no

verified medication list)		but with verification from field teams)	
ACT purchasing behavior	Whether the client purchased any kind of ACT	ACT	1: AL==1 or other_ACT==1<br>0: otherwise
After expansion of subsidized AL	Whether the exit interview took place after the list of subsidized ALs was expanded at the end of July 2021.	post_expansion	1: reg_date later than July 31, 2021<br>0: otherwise
After CHW outreach	Whether the exit interview took place after the CHW outreach was conducted at the end of September 2021.	post_CHW	1: reg_date later than September 30, 2021<br>0: otherwise
Interview date	Month and year of exit interview	reg_date	month/year

[a] *For primary analysis, ACTs will include both study approved AL and other ACTs.*
[b] *Malaria test-positive is defined as those who are mRDT+ based on the mRDT conducted at the retail outlet or who are positive by a test conducted outside of the retail outlet (either RDT or microscopy) with documented proof provided*

3.2 Secondary Outcomes

Outcome	Description	Formula to calculate outcome	Variables and Source	Specifications
Use of malaria rapid diagnostic test	Proportion of suspected malaria cases[a] that receive a malaria test<br><br>Note: We restrict to only clients tested with an mRDT at the outlet regardless of testing elsewhere. (Note that this will include those who arrived with a documented test but went on to be tested with an mRDT in the shop.)	**Numerator:** # of suspected malaria cases tested with mRDT (test=1)<br><br>**Denominator:** # of suspected malaria cases (case=1)<br><br>$\frac{\text{#suspected malaria cases tested with mRDT}}{\text{#suspected malaria cases}}$<br><br>*suspected malaria case = tested at outlet or untested and purchased AM	Raw items below	1 = suspected malaria cases tested with mRDT<br>0 = suspected malaria case not tested with mRDT
	Sensitivity analysis to restrict to clients not previously tested	**Numerator:** # of suspected malaria cases tested with mRDT not previously tested (test=1 and test_else=0)		

		**Denominator:** # of suspected malaria cases who were also not previously tested (case=1 and test_else=0)<br><br>$\frac{\text{\#suspected malaria cases tested with mRDT (not previously tested)}}{\text{\#suspected malaria cases (not previously tested)}}$<br><br>*suspected malaria case = tested at outlet or untested and purchased AM		
	Sensitivity analysis with expanded denominator, including all who consented and met inclusion criteria	**Numerator:** # of suspected malaria cases tested with mRDT (test=1)<br><br>**Denominator:** # who consented and met inclusion criteria (included=1)<br><br>$\frac{\text{\#suspected malaria cases tested with mRDT}}{\text{\#consented \& met inclusion critera}}$		
Adherence to mRDT result	Proportion of malaria tested clients whose treatment adhered to test results<br><br>This outcome is only defined on clients who were tested. Note: For primary analysis, we are interested in adherence to mRDT results for mRDTs conducted at the outlet.	**Numerator:** # of clients that tested positive with mRDT and purchased ACT + # of clients that tested negative with mRDT and did not purchase AM (purchase_anyACT_po=1 or [purchase_anyACT_ne=0 and purchase3ne=0])<br><br>**Denominator:** # of clients tested with mRDT (test=1)<br><br>$\frac{\text{number clients that tested positive with mRDT and purchased ACT + number clients that tested negative with mRDT and did not purchase AM}}{\text{number clients tested with mRDT}}$	Raw items below	1 = positive malaria test and purchased *any* ACT OR negative malaria test and did not purchase any AM<br>0 = otherwise
	As a secondary analysis, we will summarize adherence to test results from elsewhere for those who were tested elsewhere.			

Appropriate case management	Proportion of suspected malaria cases[a] that are managed appropriately.<br><br>This outcome is only defined on (i.e. calculated for) clients who were tested with an mRDT at the outlet or were untested but purchased any antimalarial. (Note: This will include those who arrived with a documented test but went on to be tested in the shop.)<br><br>Note that the numerator of this outcome is the same as for the previous outcome, namely adherence to mRDT result, but the denominator is potentially larger because it is suspected malaria cases and not only those who are tested (as for the previous outcome).	**Numerator:** # of clients that tested positive with mRDT and purchased ACT + # of clients that tested negative with mRDT and did not purchase AM (purchase_anyACT_po=1 or [purchase_anyACT_ne=0 and purchase3ne=0])<br><br>**Denominator:** # of suspected malaria cases (case=1)<br><br>$$\frac{\text{number clients that tested positive with mRDT and purchased ACT + number clients that tested negative with mRDT and did not purchase AM}}{\text{number suspected malaria cases}}$$<br><br>*suspected malaria case = tested at outlet or untested and purchased AM	Raw items below	1 = positive malaria test and purchases ACT OR negative malaria test and did not purchase any AM<br>0 = otherwise
	Sensitivity analysis with expanded denominator, including all who consented and met inclusion criteria	**Numerator:** # of clients that tested positive with mRDT and purchased ACT + # of clients that tested negative with mRDT and did not purchase AM (purchase_anyACT_po=1 or [purchase_anyACT_ne=0 and purchase3ne=0])<br><br>**Denominator:** # who consented and met inclusion criteria (included=1)<br><br>$$\frac{\text{number clients that tested positive with mRDT and purchased ACT + number clients that tested negative with mRDT and did not purchase AM}}{\text{number consented \& met inclusion critera}}$$		

ACT use among the untested	Proportion of untested clients taking ACT<br><br>The denominator is all clients who were not tested with mRDT at the outlet nor tested elsewhere.<br>The numerator is untested clients who purchased any ACT.	**Numerator:** # of untested clients who purchased ACT (purchase_anyACT_untest=1)<br>**Denominator:** # of untested clients (test=0 and test_else=0)<br>$\frac{\text{number untested clients who purchased ACT}}{\text{number untested clients}}$	Raw items below	1= purchased ACT and was not tested<br>0 = otherwise

Raw items used to calculate the secondary outcomes:

Item	Description	Variables and Source	Specifications
Test status	Did you (or your child) have your blood tested for malaria today at the outlet?	test	1 = Yes<br>0 = No
Test result	mRDT results from outlet (self-report)	test_results	1 = Negative<br>2 = Positive<br>3 = Invalid
Test elsewhere	whether a malaria test (microscopy or RDT) was performed elsewhere prior to visiting shop (self-report)	test_else	1 = Yes<br>0 = No
Test result from elsewhere	Malaria diagnostic test result before coming to the outlet (we only accept those with documentation observed)	test_else_res	1 = Negative<br>2 = Positive - report observed
Purchased AL (based on verified medication list)	Whether clients purchased any kind of AL.	AL (a new variable based on type_medXX == 1, but with verification from field teams)	1 = yes<br>0 = no
Non-AL ACT (based on verified medication list)	Whether clients purchased non-AL ACT. [1]	other_ACT (a new variable based on type_medXX == 2, but with verification from field teams)	1 = yes<br>0 = no
Other antimalarial	Whether clients purchased other antimalarial.	other_AM (a new variable based on type_medXX == 3,	1 = yes<br>0 = no

[1] Note re framing: frame this as clients who purchased an ACT other than the primary ACT in the national guidelines because some other ACTs are equally effective compared to AL, and we would like to ensure the study results are valuable in other settings where other drugs are more prominent (e.g. DRC where ASAQ and DHA-PQP are likely more prominent (for now).

		but with verification from field teams)	
Antimalarial purchasing behavior	Whether the client purchased any antimalarial	AM	1: AL ==1 or other_ACT==1 or other_AM==1<br>0: otherwise
Suspected malaria cases	Whether a client is a suspected malaria case	case	1: test==1 or AL ==1 or other_ACT==1 or other_AM==1<br>0: otherwise
Purchased other AM with negative test	Whether a client purchased non-ACT AM and had negative mRDT test result	(new variable based on other_AM and test_results) purchase3ne	1: test_results==1 and (other_AM==1)<br>0: otherwise
Purchased ACT with positive test	Whether a client purchased any ACT and had positive test mRDT result	(new variable based on AL, other_ACT, and test_results) purchase_anyACT_po	1: test_results==2 and (AL==1 or other_ACT==1)<br>0: otherwise
Purchased ACT with negative test	Whether a client purchased any ACT and had negative test mRDT result	(new variable based on AL, other_ACT, and test_results) purchase_anyACT_ne	1: test_results==1 and (AL==1 or other_ACT==1)<br>0: otherwise
Purchased ACT untested	Whether a client purchased any ACT and was untested (neither mRDT at shop, nor elsewhere)	(new variable based on AL, other_ACT, and test_results) purchase_anyACT_untest	1: (test ==0 and test_else ==0) and (AL==1 or other_ACT==1)<br>0: otherwise

[a] *"Suspected malaria case": any client (*with fever, or history of fever in the last 48 hours, or suspects they may have malaria) *who was tested with an mRDT at the outlet (this will include those who arrived with a documented test but went on to be tested in the shop) or was untested but purchased any antimalarial (AM)*

3.3 Additional Variables of Interest

Variable	Description	Variables and Source	Specifications
Respondent gender	Gender of the respondent	respondent_gender	1: Female<br>0: Male

Child gender	Gender of the child, if suspected case is a child	child_gender	1: Female<br>0: Male
Client[2] gender	Gender of the respondent, or of child, if suspected case is a child	respondent_gender if respondent= "Adult with fever"; child_gender if respondent= "Guardian of the child"	1: Female<br>0: Male
Respondent age	Respondent's age	respondent_age	0: 0-17<br>1: 18-25<br>2: 26-39<br>3: 40-59<br>4: 60-79<br>5: 80+
Child age	Child's age, if suspected case is a child	child_age	0: 0-17<br>1: 18-25<br>2: 26-39<br>3: 40-59<br>4: 60-79<br>5: 80+
Client age	Age of the respondent, or of child, if suspected case is a child	respondent_age if respondent= "Adult with fever"; child_age if respondent= "Guardian of the child"	0: 0-17<br>1: 18-25<br>2: 26-39<br>3: 40-59<br>4: 60-79<br>5: 80+
Household size			Continuous
Highest level of education completed	What is the highest level of schooling you completed?	school	1: None<br>2: Primary<br>3: Secondary<br>4: College<br>5: University

[2] Where client refers to the suspected malaria case

Occupation	What is your primary occupation?	occupation	1: Farming/Livestock keeping<br>2: Government or parastatal<br>3: Private Sector<br>4: Self-employed (with employees)<br>5: Self-employed (without employees e.g. motorcycle taxi, vendor)<br>6: Unpaid family helper in a business<br>7: Casual worker/day laborer<br>8: House help<br>9: Homemaker<br>10: Student<br>11: Not available to work<br>555: Other
Wealth category			Binary: under poorest 40th centile or not
Seriousness of disease	How serious is this illness in your opinion?	serious	1: Not very serious/minor<br>2: Moderate<br>3: Very serious
Duration of symptoms	How many days ago did the symptoms start?	symptoms_start	1: within the last 48 hours<br>2: 3-4 days<br>3: 5-7 days<br>4: 14 days/2 weeks<br>5: 21 days/3 weeks<br>6: 30 days/one month<br>7: Longer than a month<br>555: Don't know

3.4 Summary of Numerators and Denominators for Primary and Secondary Outcomes

Outcome	Numerator	Denominator
**Primary outcome**		
**ACT purchased by test positive**	tested[3] positive and purchased ACT	purchased ACT
Sensitivity analyses		
1. Restrict to AL (i.e. any AL)	tested positive and purchased AL	purchased AL
2. Timing of expansion of subsidized ALs	tested positive and purchased ACT after July 31, 2021	purchased ACT after July 31, 2021
3. Timing of CHWs, other outreach	tested positive and purchased ACT after Sept 30, 2021	purchased ACT after Sept 30, 2021
**Secondary outcomes**		
**Use of mRDT**	mRDT tested	suspected malaria[4]
Sensitivity analyses		
1. Use of mRDT with no prior test	mRDT tested (and not previously tested elsewhere)	suspected malaria (not previously tested)
2. Use of mRDT (expanded denominator)	mRDT tested	consented & met inclusion criteria
**Adherence to mRDT result**	(mRDT+ and ACT) OR (mRDT- and no AM)	tested
**Appropriate case management**	(mRDT+ and ACT) OR (mRDT- and no AM)	suspected malaria
1. Sens. analysis - Expanded denominator	(mRDT+ and ACT) OR (mRDT- and no AM)	consented & met inclusion criteria
**ACT use among untested**	purchased ACT and untested[5]	untested

[3] Any test, either at the outlet or prior to visiting outlet (but with documentation)
[4] Suspect malaria: tested at outlet or untested and purchased AM
[5] With no test at outlet or prior to visiting outlet

4 Statistical Analysis Plan

4.1 Demographic and Clinical Characteristics ("Table 1")

Descriptive statistics for continuous variables will include the mean, standard deviation, median, range and the number of observations. Categorical variables will be presented as numbers and percentages. No significance tests will be performed to test for differences at baseline. We will use principal components analysis to define a wealth index. Specifically, we will use polychoric principal components analysis using responses to the wealth index questions using the same approach as outlined in Supplement S1 of O'Meara et al (9). For more details, see section below.

4.2 Intervention Characteristics

We will utilize the outlet-reported data collected by the TESTsmART App to summarize the intervention characteristics, including the total number of suspected malaria fevers, total number of mRDTs performed in the outlets, total number of ACTs distributed, total payment the outlets received, rates of activities and positive malaria cases, etc. These outlet-level characteristics will be summarized descriptively, and potentially by arm and season. Caution is needed due to the fact that the App data will be reported by the outlets and considerable bias may be introduced by such data collection method. Consequently, we may choose to exclude these results from the final manuscript. Exit interview data will be used to provide further insight by providing summaries of reported intervention implementation (e.g. RDT taken and free ACT received) which could be compared to the summary statistics on implementation reported in the app. We note too that the intervention was reinforced in September 2021 through a range of strategies (e.g. for CD outlets, by raising awareness of the availability of free ACTs conditional on a positive mRDT) and therefore we would expect some greater implementation of the client-directed intervention following these changes.

4.3 Analyses

General modeling principles. We will analyze client-level self-reported outcomes using the modified Poisson approach (4,5), with log link to estimate risk ratios (RRs) and identity link to estimate risk differences (RDs). Such an approach assumes a Poisson distribution for the binary outcome and then 'fixes' the estimated standard errors via the sandwich variance estimator to obtain 'robust' SEs in order to correct for model misspecification. We note that the modified Poisson approach is implemented within the generalized estimating equations (GEE) (6,7) framework with, as noted above, robust standard errors. This is also our preferred framework with which to account for clustering of outcomes by outlet. Moreover, as noted below, the robust standard errors will be further adjusted for potential "small-sample" bias due to the fact that there are fewer than 50 clusters.

Mean model. The outcome will be regressed on two binary indicators for each of Control and CD, with treatment arm PD+CD (the combined interventions) serving as the reference group in order to facilitate estimation of the two key contrasts of interest, namely:

1. Control vs. CD+PD
2. CD vs. CD+PD

The mean model will also include fixed effects for the stratification variables, and in sensitivity analysis, will include adjustment for a vector of potential confounder variables (see details below) to account for possible imbalances between study arms.

The mean models will have the following forms (noting that the combined intervention arm, PD+CD, is the reference level since it appears in both key contrasts of interest):

Model 1: $\log\left(E\left(Y_{ij}\right)\right) = \beta_0 + \beta_1(Control)_{ij} + \beta_2 CD_{ij} + \boldsymbol{s_{ij}}'\boldsymbol{\gamma} + \boldsymbol{w_{ij}}'\boldsymbol{\alpha} + \boldsymbol{t_{ij}}'\boldsymbol{b}$

Model 2: $E\left(Y_{ij}\right) = \beta_0 + \beta_1(Control)_{ij} + \beta_2 CD_{ij} + \boldsymbol{s_{ij}}'\boldsymbol{\gamma} + \boldsymbol{w_{ij}}'\boldsymbol{\alpha} + \boldsymbol{t_{ij}}'\boldsymbol{b}$

where Y_{ij} is the primary outcome whether client j in outlet i (j=1,…,n_i; i = 1…, 39, where n_i is the number of eligible clients in outlet i) who purchases ACT is malaria test positive or not (=1 if test positive, 0 untested or test negative) and $E\left(Y_{ij}\right)$ is its expected value. $(Control)_{ij}$ is an indicator for whether the client is in an outlet allocated to the control arm (=1 if the client is assigned to control, =0 otherwise) and CD_{ij} is an indicator for whether the client is in an outlet allocated to the CD treatment arm (=1 if the client is assigned to the CD arm, =0 otherwise). $\boldsymbol{s_{ij}}$ is a vector of stratification indicators and $\mathbf{w}_{ij}$ is a vector of potential confounding variables (e.g., age, gender, wealth, education) to account for possible imbalances between study arms, where we note $\boldsymbol{s_{ij}}$ will be included in all analyses(8), whereas $\mathbf{w}_{ij}$ will be included in sensitivity analyses. In all analyses, we will also include a vector of adjustors for time ($\boldsymbol{t_{ij}}$) to control for potential confounding by time (which could arise if the rate of data accrual over time is not the same between arms, e.g. due to varying client volume,) and to increase precision of the treatment effect. We will model time flexibly by specifying $\boldsymbol{t_{ij}}$ as a vector of dummy variables indicating blocks of time (e.g., 2-3 months).

Our primary set of adjustment variables is planned to be the same as those used by O'Meara et al (9), specifically gender and age of the client (categorized as <5, 5-17, or 18+ years), occupation and education level of the client or guardian (if client <18 years), household size and wealth index (categorized in to quintiles, or a coarser split such as lower 40% vs. upper 60%, as needed to facilitate model convergence; see details of the calculation of wealth index below in the last section prior to Section 5). Additionally, in the case that there are marked differences in baseline outlet-level characteristics, additional adjustment will be made. For example, we expect variability in characteristics such as baseline client volume or willingness to test (e.g. based on app data) and will additionally consider adjustment for relevant variables.

Given that the literature indicates that when there are fewer than 50 clusters in a CRT, small sample correction methods should be used to ensure that standard error estimates are correctly estimated when using GEE to analyze binary outcomes, and given that the size of the CRT is close to this cut-off, we plan to adopt the use of the Kauerman-Carroll correction if coefficient of variation of cluster size is smaller than 0.6 and Fay and Graubard correction otherwise, to avoid any possible problems (11,12). Because all secondary outcomes are binary, we will use the same modelling approach to compare these outcomes.

(Note on dummy coding vs. effect coding. As per the assumptions used in our sample size calculations for the four-arm designs (see section 2.6), we anticipated there would be an interaction effect between the provider-focused and the client-focused incentives. As such, our primary research questions focus on comparisons between specific trial arms, and we therefore planned a model that explicitly parameterized all four individual arms, rather than parameterizing with an interaction term. We had also considered using

effect coding rather than the dummy coding formulation shown above but given that main effects were not of primary interest and the interaction term is not explicitly modelled, the orthogonality property that comes with effect coding would not provide additional benefit over dummy coding. Therefore, we will use dummy coding for its readily interpretable parameters that are aligned with our contrasts of interest.)

Our primary goals are to determine whether the proportion of ACT sold to malaria test positive clients increases significantly in the PD+CD arm as compared to the Control and CD arms. These can be evaluated by testing the null hypotheses H_0: $\beta_1=0$ and H_0: $\beta_2=0$, using the Benjamini Hochberg procedure to control the false discovery rate at 5%. For secondary outcomes, we will not report p-values but confidence intervals for the intervention effects.

**Table 5: Contrasts of interest**	**Population**	**Contrast**	**Error Control**
**Primary outcomes**			**False Discovery Rate**
(a) Effect of PD+CD on the proportion of ACT sold to mRDT positive, compared to the Control arm	All clients who purchased ACT	$-\beta_1$	0.05
(b) Effect of PD+CD on the proportion of ACT sold to mRDT positive, compared to the CD arm	All clients who purchased ACT	$-\beta_2$	

As mentioned before, in addition to providing risk ratios, we will also provide estimates of absolute effects (risk differences) in order to provide an intuitive measure of the potential public benefit of the interventions. Such an approach is recommended by the CONSORT statement on reporting of cluster randomized trials (13). These risk differences will be estimated by changing the log-link to an identity link (i.e. Model 2 rather than Model 1).

In the unlikely case that the modified Poisson models fail to converge, we will use the following strategy:

- If convergence fails for the modified Poisson model with log link (i.e. that used to estimate risk ratios)
  - A log-link with binomial family will be used.
  - If that fails, marginal standardization will be used to derive risk ratios from odds ratios estimated by using the standard logit link for binomial outcomes.
- If convergence fails for the modified Poisson model with identity link (i.e. that used to estimate risk differences), which is a likely occurrence for at least one of the outcomes:
  - We will assume a binomial distribution with identity link together with robust standard errors, where we note that the binomial approach is expected to converge less often than the modified Poisson approach but it is still important to assess before moving to the next step.
  - If that fails, we will assume a normal distribution with identity link together with robust standard errors to correct for model misspecification.
  - If that fails, marginal standardization will be used to derive risk differences from odds ratios estimated by using the standard logit link for binomial outcomes.

Correlation model. We plan to use a second set of estimating equations to estimate the correlation parameters. Specifically, we will use the matrix-adjusted estimating equation (MAEE) approach (15)

rather than the standard method of moments approach typically used in GEE. Together, we refer to GEE combined with MAEE as the paired GEE/MAEE approach. By using a second set of estimating equations, we will be able to directly estimate the correlation parameters as well as corresponding 95% confidence intervals. Moreover, by using MAEE as the second set of estimating equations, we are able to minimize the finite-sample bias expected for the correlation parameters themselves. Additionally, by applying finite-sample corrections to the standard errors, we can obtain confidence intervals with better coverage.

Given the long time period over which participants are recruited into the study, and our expectation that pairwise correlations between outcomes in participants in the same outlet (i.e. cluster) will decay over time, our base correlation structure will be an exponential decay correlation structure. To model this, follow-up time will be split in to "periods" such as month or quarter or some other appropriate scale. We note that these periods will likely, though not necessarily, be of the same structure as that used to model time in marginal mean models (1) and (2). Given prior experience analyzing longitudinal CRTs, we expect that there may be challenges with achieving model convergence with the exponential decay model. If so, we will next consider a nested exchangeable structure, followed by the traditional exchangeable correlation structure. Furthermore, we will consider allowing different degrees of correlation in each arm of the trial. To provide further insight as to the appropriateness of the chosen correlation structure, we will report the correlation information criterion (CIC) (16) measure for each of the structures, noting that there is still a need to determine the most appropriate model fit criteria for correlation structures. We note that we do not expect sensitivity of our intervention effect estimates (e.g. from models (1) and (2) above) to parameterization of the correlation structure as a result of the "robustness" properties of the GEE approach to estimation of the mean model parameters. Instead, as noted above, our primary reason for carefully and correctly specifying the correlation structure is to facilitate reporting of correlation parameters. Standard errors of the correlation parameters will be estimated using a small-sample corrected sandwich variance estimator. More specifically, we expect to use the Mancel-DeRouen correction based on prior simulation work (15). In the case that more recent methodological work indicates that an alternative method is expected to perform better, we will adjust our strategy.

If the MAEE approach will not converge, we will revert to the GEE approach with method of moments estimation of correlation parameters, and note that inference on the parameters of the mean model, including intervention effects, is expected to be the same whether or not MAEE is used.

Inference. Inference for parameters of the mean model and of the correlation model will be based on the t-statistic with degrees of freedom I-p and I-q, for the mean and correlation models respectively, where p and q are the number of parameters in the mean and correlation models, respectively (specifically of the cluster-level covariates, including the intercept). Moreover, small-sample correction will be applied to the sandwich variance estimates of SE of the parameters of each model, as outlined in the relevant sections above.

Compliance. All analyses will be based on the intention-to-treat principle whereby all clients will be included in the analysis irrespective of whether they complied with the intervention in the outlet at which they sought care (e.g. even if they did not use the ACT subsidy if they tested positive in an outlet in CD and PD+CD that received the client-directed intervention). We note that compliance in this context would be complex to define (at both shop level and participant level).

Missing/incomplete data. Since we do not have longitudinal follow-up, we will not need to account for missing data due to attrition of clients. If possible, patterns of incomplete data will be described and compared by outlet and between arms from the following perspectives:

- When interviewers cannot or do not go to the shop (e.g. social unrest preventing visits to shops);
- Clients refuse to participate in the exit interview;
- Interviews stop halfway through.

Moreover, if some questions are missing on the exit interview, a table such as Table B7 will enable us to better assess possible issues.

Further considerations related to cluster size and rate of recruitment over time. Although we target an equal number of exit interviews per outlet and target constant recruitment over time, we recognize that the nature of client flow and timing of exit interviews may lead to variability in cluster size and/or differential rates of accrual of exit interviews over time. In the case that variable cluster size occurs, we expect that the variability in cluster size will be mostly related to underlying client flow and, as such, we do not expect to adjust our primary analyses. In this case, we will consider sensitivity analyses with weighting by cluster size so as to assess sensitivity to the observed variability, whilst noting that the target estimand that corresponds to such weighting is different to that without weighting [17, 18]. In the case that the rate of recruitment changes considerably over time and is different between outlets, we will be concerned about the potential for confounding by time, even in the context of the parallel-arm randomized trial design. As a consequence, we have pre-specified our primary analysis to adjust for calendar time, (e.g. in the flavor of approaches used in the analysis of stepped-wedge cluster randomized trials) in order to protect against such potential confounding. We have selected this strategy as, even in the case that there is no confounding by time due to differing rates of data accrual, such an approach is expected to produce unbiased intervention effect estimates and would lead to increased precision of those intervention effects in the case that time is predictive of outcome levels. We recognize that the potential increase in precision may not fully compensate for the additional penalty to degrees of freedom imposed by adjustment for time. Nevertheless, such an approach is a fair tradeoff as it is important that the primary analysis be planned to minimize bias in intervention effect estimation.

Further considerations related to conditional outcomes (i.e. related to outcome definitions). Given that our outcome definitions restrict which individuals will be included in a given analysis, there is the possibility of lack of exchangeability between arms for the sub-populations included in analysis of a given outcome. A priori, we do not expect lack of exchangeability but will carefully examine covariate descriptive statistics between treatment arms for the sub-populations of individuals included in analysis of a given outcome (e.g. see draft tables B1.1-B1.4). In the case that considerable imbalance is observed for a given analysis, analyses with covariate adjustment (i.e. the full mean model with adjustment for $\boldsymbol{w_{ij}}$) may be considered more appropriate than those without such covariate adjustment.

Further considerations related to the time required to perform RDT testing and to participate in the exit interview. Additional potential issues in analysis could be related to the fact that clients need time in order to participate in testing with RDT and to participate in the exit interview of the study. We have developed a working version of a directed acyclic graph. We note that, to date, the current form of the DAG has not provided us with other strategies for analysis but that it is valuable to document possible mechanisms by which we may expect to see a sample in the exit interview that is not fully reflective of the

underlying population or by which the CD intervention may be less appealing to certain kinds of individuals who have less time available to undertake testing by RDT.

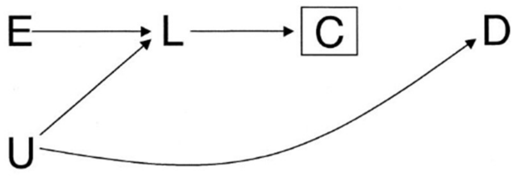

To this end, denote E as the exposure, C as the indicator for whether the client participated in the exit interview, D as the outcome (proportion of ACT sold to mRDT positive). L is whether the client purchased mRDT, U can be beliefs in mRDT results or willingness to purchase mRDT etc. We anticipate L to have an effect on C, because the mRDT will take 15 to 20 mins and the exit interview will take additional time. By restricting the analysis to the samples we can obtain, C, the following sources of selection bias may arise:

- When clients have purchased an mRDT and spent time waiting for the test results, they can become less willing to participate in the exit interview.
- If a client is aware of mRDT and comes to the outlet ready to take a test, that implies the client is more likely to have enough time and can increase the probability of participating in the exit interview.
- Clients who are impatient to take the mRDT may also be impatient to take the exit interview

However, data on clients unwilling to participate in the exit interview will be largely unavailable. To partly investigate the issue of selection bias, field team will try to document and describe different reasons and patterns of refusal to participation according to season, malaria transmission intensity, geographic area, child/adult client, etc. As noted above, the DAG here and the issues outlined here may arise and present challenges to analysis for which we currently have no proposed strategies.

Further information regarding creation of a wealth index[6] Variables used and methods included to create the wealth index will be based on methods developed by Demographic and Health Surveys (DHS) (19). Frequency tables will be created for each of the indicators in the wealth index. Sparsity in categories will be improved by collapsing categories into meaningful dimensions (e.g. in prior study, wall materials was grouped into "porous" and "non-porous"; roof type into thatched vs. not). Once meaningful categories are created for each of the categorical variables, binary indicators will be created to represent each "category". For all index variables, the binary value of one will correspond to the item associated with higher SES. Because our variables are categorical, rather than the normally distributed continuous variables appropriate for the default Pearson correlation matrix constructed as part of traditional principal components analysis (PCA) analysis, a polychoric correlation matrix will be computed for the final set of variables and used in the subsequent factor analysis, retaining only the first factor (20). To complete the wealth index, a factor score will be computed for each household. Construction of the wealth index will be performed using the polychoricpca package (21) in Stata 15 SE. In practice, we expect to parameterize the index in wealth quintiles as in O'Meara et al (9).

5 Addendum for Additional Analyses

Note: For sensitivity analyses, we will follow the same approach as for the primary outcome, using GEE/MAEE but reverting to a standard GEE approach if GEE/MAEE does not converge or is prohibitively

[6] Note that the text here is copied almost verbatim from Supplement S1 of O'Meara et al (9).

time consuming to run. We note that there is expected to be high computational burden for these analyses and therefore we do expect to revert to standard GEE.

See Section 3.4 for a summary of all sensitivity analyses for both the primary and secondary outcomes.

5.1 Sensitivity analyses for primary outcome

5.1.1 Restricted to ALs

For this sensitivity analysis, the models will be the same as in section 4, with the exception that the outcome will be defined only for clients who purchased an AL, as opposed to any ACT.

5.1.2 Expansion of subsidized ALs

For this sensitivity analysis, we will include an indicator of whether a client was interviewed before or after the expansion of ALs that qualified for subsidy. The models from section 4 will be modified as follows:

$$\text{Model 1: } \log\left(E(Y_{ij})\right) = \beta_0 + \beta_1(Control)_{ij} + \beta_2 CD_{ij} + \beta_3 EAL_{ij} + \beta_4 EAL_{ij} * (Control)_{ij} + \beta_5 EAL_{ij} * CD_{ij} + \boldsymbol{s_{ij}}'\boldsymbol{\gamma} + \boldsymbol{w_{ij}}'\boldsymbol{\alpha} + \boldsymbol{t_{ij}}'\boldsymbol{b}$$

$$\text{Model 2: } E(Y_{ij}) = \beta_0 + \beta_1(Control)_{ij} + \beta_2 CD_{ij} + \beta_3 EAL_{ij} + \beta_4 EAL_{ij} * (Control)_{ij} + \beta_5 EAL_{ij} * CD_{ij} + \boldsymbol{s_{ij}}'\boldsymbol{\gamma} + \boldsymbol{w_{ij}}'\boldsymbol{\alpha} + \boldsymbol{t_{ij}}'\boldsymbol{b}$$

where EAL_{ij} is an indicator of whether client j in outlet i was interviewed after expansion of the AL subsidy (end of July 2021). All other terms are defined as in section 4.

5.1.3 Pre- vs. post-CHW deployment

For this sensitivity analysis, we will include an indicator of whether a client was interviewed before or after the CHW deployment. The models from section 4 will be modified as follows:

$$\text{Model 1: } \log\left(E(Y_{ij})\right) = \beta_0 + \beta_1(Control)_{ij} + \beta_2 CD_{ij} + \beta_3 CHW_{ij} + \beta_4 CHW_{ij} * (Control)_{ij} + \beta_5 CHW_{ij} * CD_{ij} + \boldsymbol{s_{ij}}'\boldsymbol{\gamma} + \boldsymbol{w_{ij}}'\boldsymbol{\alpha} + \boldsymbol{t_{ij}}'\boldsymbol{b}$$

$$\text{Model 2: } E(Y_{ij}) = \beta_0 + \beta_1(Control)_{ij} + \beta_2 CD_{ij} + \beta_3 CHW_{ij} + \beta_4 CHW_{ij} * (Control)_{ij} + \beta_5 CHW_{ij} * CD_{ij} + \boldsymbol{s_{ij}}'\boldsymbol{\gamma} + \boldsymbol{w_{ij}}'\boldsymbol{\alpha} + \boldsymbol{t_{ij}}'\boldsymbol{b}$$

where CHW_{ij} is an indicator of whether client j in outlet i was interviewed after CHW deployment (end of Sept 2021). All other terms are defined as in section 4.

5.2 Sensitivity analyses for secondary outcomes

5.2.1 For secondary outcome "use of mRDT" – restricted to clients not previously tested

For this sensitivity analysis, the outcome will be defined only for clients who came to the shop without a prior test from elsewhere.

5.2.2 For secondary outcome “use of mRDT” - with expanded denominator

For this sensitivity analysis, the denominator will be all clients who consented and met all inclusion criteria.

5.2.3 For secondary outcome “appropriate case management” - with expanded denominator

For this sensitivity analysis, the denominator will be all clients who consented and met all inclusion criteria.

6 Appendix

Appendix A1: CONSORT flow-chart for progress of clusters and individuals through intervention arms

See example figure from Coupon study copied here, where we note that we do not formally distinguish between follow-up time periods but, instead collect data over the whole follow-up period. As such, we could present the data for all follow-up time points in a single combined box but there are also benefits to showing data accrual by time period when there is such a long follow-up period.

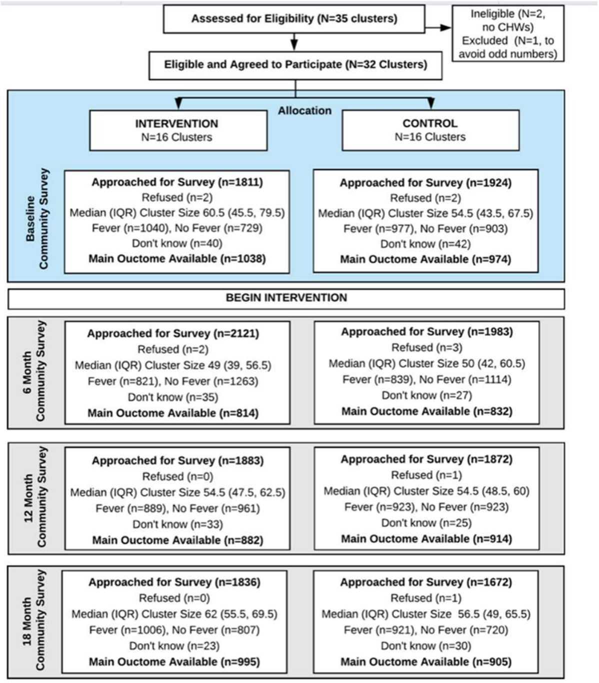

Table A2. Sample Size by Time Period– n (%), unless otherwise noted

Study Arm	Time Period			
	Period 1	Period 2	Period 3	Period 4
**Exit Interviews conducted**				
Control				
CD				
CD+PD				
**Included and Consented**				
Control				
CD				
CD+PD				
**Suspected Malaria Cases**[a]				
Control				
CD				
CD+PD				
**Purchased ACT**				
Control				
CD				
CD+PD				
**Tested with mRDT at outlet**				
Control				
CD				
CD+PD				
**Untested clients taking ACT**				
Control				
CD				
CD+PD				

[a] *"Suspected malaria case": any client (*with fever, or history of fever in the last 48 hours, or suspects they may have malaria) *who was tested with an mRDT at the outlet (this will include those who arrived with a documented test but went on to be tested in the shop) or was untested but purchased any antimalarial (AM)*

Appendix A3: TimelineCluster illustration of blinding by intervention arm (using original four arms for illustrative purposes)

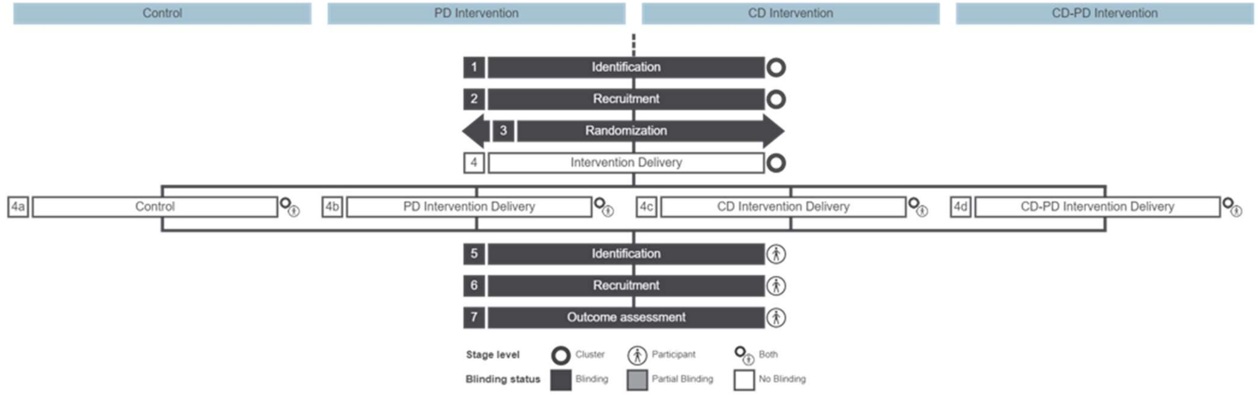

1 Identification
In each country, among those private registered retail outlets that expressed interest in participating in the study, eligible private outlets were identified according to set criteria.

2 Recruitment
In Kenya, a full sampling frame of eligible shops was generated in each county and outlets were randomly selected for enrollment from the sampling frame. If a shop declined enrollment, they were replaced with one of the remaining shops. In Nigeria, a complete listing of PPMVs was stratified on three geographic regions of the city and shops were enrolled from a random subset proportional to the size of the strata.

3 Randomization
Following enrollment, the retail outlets were randomized to arms separately within each country by the study statistician.

4 Intervention Delivery
Due to the nature of the interventions, it is not possible to blind participants and the implementation team to the study arm allocated to each outlet.

4a Control
Control intervention: mRDTs are made available at wholesale price to the retail outlet, and outlet owner/attendant is trained to use mobile reporting app

4b PD Intervention Delivery
Provider-directed (PD) intervention: in addition to the interventions implemented in the control outlets, the retail outlet owner in this arm receives a small incentive to perform the mRDT (approximately USD $0.10 for each mRDT test they report using the mobile app). In Kenya, the PD intervention will be excluded due to sample size considerations

4c CD Intervention Delivery
Client-directed (CD) intervention: in addition to the interventions implemented in the control outlets, the clients visiting outlets in this arm receive a free ACT (cost equivalent to 150 Kenyan Shillings(KES) for adults and 60 KES for children; ENTER NIGERIA pricing) if they purchase an mRDT and have a positive test result(conditional subsidy)

4d CD-PD Intervention Delivery
Combined (CD-PD) intervention: in addition to the interventions implemented in the control outlets, retail outlet owners in this arm receive an incentive to test for malaria and clients visiting these outlets receive an a free ACT discount conditional on a positive test result (i.e. this arm is a combination of the PD and CD interventions)

5 Identification
Data collectors will be blinded throughout collection. All clients exiting the shop that day are eligible to be screened and field researchers are instructed to make no pre-judgements about clients but rather approach the next available client exiting the shop.

6 Recruitment
Interviewees must meet specific eligibility criteria to proceed with the exit interview. The study team will conduct a verbal consent process for participants who meet the inclusion/exclusion criteria, prior to participation in the exit interview outside retail outlets.

7 Outcome assessment
Interviewers collecting the outcome data will be blinded to allocation status.

Appendix B: Shell tables for main analysis

Table B1.1[a]. Sample characteristics by intervention arm of all participants in the exit interview[b] – n (%), unless otherwise noted

	Control	CD	CD+PD	All Arms Combined
**Demographic Characteristics**				
All individuals in the exit interview[b]				
Adult				
Child				
Age (respondent)				
Gender (respondent)				
Relationship (respondent)				
Age (years) – mean (SD)				
Gender				
**Socioeconomic Status**				
Household size				
Highest level of schooling				
Proxy Respondent for Child				
Adult				
Occupation Category (???)				
Number of animals				
Roof Type				
Owns Land				
Wealth quintile				
Lowest				
Low				
Middle				
High				
Highest				
**Period of data collection**				
Q1				
Q2				
Q3				

**Outlet Characteristics**
**?**
**?**

[a] This table will be repeated for each time period.

[b] Includes only interviewees who consented and met all inclusion criteria.

Table B1.2. Sample characteristics by intervention arm, stratified by taking ACT vs. not taking ACT – n (%), unless otherwise noted.

Note that this table enables us to compare between study arms characteristics of those in the primary outcome analysis i.e. for those who purchased an ACT

	Taking ACT				Not Taking ACT			
	Control	CD	CD+PD	All Groups	All Groups	Control	CD	CD+PD
**Demographic Characteristics**								
Febrile individual								
Adult								
Child								
Age (respondent)								
Gender (respondent)								
Relationship (respondent)								
Age								
Gender								
**Socioeconomic Status**								
Household size								
Highest level of schooling								
Proxy Respondent for Child								
Adult								
Occupation Category (???)								
Number of animals								
Roof Type								
Owns Land								

Table B1.3. Sample characteristics by intervention arm, stratified by suspected malaria cases – n (%), unless otherwise noted Note that this table enables us to compare between study arms characteristics of those in the analysis of the secondary outcome "use of mRDT " i.e. for those who are considered a suspected malaria case

	Suspected Malaria Cases				Not Suspected Malaria Cases			
	Control	CD	CD+PD	All Groups	All Groups	Control	CD	CD+PD
**Demographic Characteristics**								
Febrile individual								
Adult								
Child								
Age (respondent)								
Gender (respondent)								
Relationship (respondent)								
Age								
Gender								
**Socioeconomic Status**								
Household size								
Highest level of schooling								
Proxy Respondent for Child								
Adult								
Occupation Category (???)								
Number of animals								
Roof Type								
Owns Land								

Table B1.4. Sample characteristics by intervention arm, stratified by mRDT performed – n (%), unless otherwise noted. Note that this table enables us to compare between study arms characteristics of those in the analysis of the secondary outcome "adherence to mRDT result " i.e. for those who reported having an mRDT performed at the outlet

	mRDT performed				No mRDT performed			
	Control	CD	CD+PD	All Groups	All Groups	Control	CD	CD+PD
**Demographic Characteristics**								
Febrile individual								
Adult								
Child								
Age (respondent)								
Gender (respondent)								
Relationship (respondent)								
Age								
Gender								
**Socioeconomic Status**								
Household size								
Highest level of schooling								
Proxy Respondent for Child								
Adult								
Occupation Category (???)								
Number of animals								
Roof Type								
Owns Land								

Table B2.1. Sample Proportions for Testing and Treating Outcomes and Behavior

	Control	CD	CD+PD	All Groups
**Testing Behavior**				
**Had mRDT in shop**				
Yes				
Positive				
ACT				
No ACT				
Negative				
ACT				
No ACT				
No				
ACT				
No ACT				
**Documented test prior to coming to shop with no mRDT in shop**				
Yes				
Positive				
ACT				
No ACT				
Negative				
ACT				
No ACT				
No				
ACT				
No ACT				

Table B2.2. Sample proportions for primary and secondary outcomes by arm

	Intervention Arms - % (n/N)		
**Outcomes**	**CD**	**CD+PD**	**Control**
**Primary Outcome**			
ACTs sold to malaria test-positive clients (N= ??)			
**Secondary Outcomes**			
Suspected malaria cases that receive a malaria test (N=??)			
Malaria tested clients whose treatment adhered to test results (N= ??)			
Suspected malaria cases that are managed appropriately (N= ??)			
Untested clients taking ACT (N= ??)			

Table B2.3. Sample proportions for sensitivity analyses by arm

	Intervention Arms - % (n/N)		
**Outcomes**	**CD**	**CD+PD**	**Control**
**Primary Outcome**			
**Sensitivity 1 – AL instead of ACT**			
ALs sold to malaria test-positive clients			
**Sensitivity 2 – timing of expansion of subsidized ALS**			
ACTs sold to malaria test-positive clients before expansion			
ACTs sold to malaria test-positive clients after expansion			
**Sensitivity 3 – timing of CHW and other outreach**			
ACTs sold to malaria test-positive clients before CHW outreach			
ACTs sold to malaria test-positive clients after CHW outreach			
**Secondary Outcomes**			
Suspected malaria cases that receive a malaria test			
**Sensitivity 1 – No prior test**			
Suspected malaria cases that receive a malaria test with no prior test			
**Sensitivity 2 – Expanded denominator**			
Consented and met inclusion criteria that receive a malaria test			
Suspected malaria cases that are managed appropriately			
**Sensitivity 1 – Expanded denominator**			
Consented and met inclusion criteria that are managed appropriately			

Table B3.1 Analyses of primary outcome

Outcomes	Risk Ratio		Risk Difference	
	Minimally[7] adjusted estimate (95% CI)	Fully adjusted estimate (95% CI)	Minimally adjusted estimate (95% CI)	Fully adjusted estimate (95% CI)
**Primary Outcome**				
ACTs sold to malaria test-positive clients (N= ??)				
CD+PD arm compared to Control arm				
CD+PD arm compared to CD arm				
**Sensitivity 1 – AL instead of ACT**				
ALs sold to malaria test-positive clients (N= ??)				
CD+PD arm compared to Control arm				
CD+PD arm compared to CD arm				
**Sensitivity 2 – timing of expansion of subsidized ALs[a]**				
ACTs sold to malaria test-positive clients (N= ??)				
CD+PD arm compared to Control arm before expansion				
CD+PD arm compared to CD arm before expansion				
CD+PD arm compared to Control arm after expansion				
CD+PD arm compared to CD arm before expansion				
**Sensitivity 3 – timing of CHW and other outreach[b]**				
ACTs sold to malaria test-positive clients (N= ??)				
CD+PD arm compared to Control arm before CHW outreach				
CD+PD arm compared to CD arm before CHW outreach				
CD+PD arm compared to Control arm after CHW outreach				
CD+PD arm compared to CD arm before CHW outreach				

[a] p-value for CD+PD vs. CD for before vs. after = ?; p-value for CD+PD vs. control for before vs. after = ?; [b] p-value for CD+PD vs. CD for before vs. after = ?; p-value for CD+PD vs. control for before vs. after = ?

Note that we will additionally provide estimated time effects and correlation parameters for the main analyses of the primary outcome. We expect to include it in the footnote to this table – or will expand to a table if we see considerable differences in estimates between different models.

Note also that we plan to provide visualizations of these analyses in the form of forest plots.

[7] Minimally adjusted: adjusted only for design variables i.e. strata; fully adjusted: additionally adjusted for pre-specified individual-level and cluster-level variables.

Table B3.2 Analyses of secondary outcomes

Outcomes	Risk Ratio<br>Minimally[8] adjusted estimate (95% CI)	Risk Ratio<br>Fully adjusted estimate (95% CI)	Risk Difference<br>Minimally adjusted estimate (95% CI)	Risk Difference<br>Fully adjusted estimate (95% CI)
**Suspected malaria cases that receive a malaria test (N=??)**				
CD+PD arm compared to Control arm				
CD+PD arm compared to CD arm				
**Sensitivity 1 – No prior test**				
Suspected malaria cases that receive a malaria test with no prior test (N=??)				
CD+PD arm compared to Control arm				
CD+PD arm compared to CD arm				
**Sensitivity 2 – Expanded denominator**				
Consented and met inclusion criteria that receive a malaria test (N=??)				
CD+PD arm compared to Control arm				
CD+PD arm compared to CD arm				
**Malaria tested clients whose treatment adhered to test results**				
CD+PD arm compared to Control arm				
CD+PD arm compared to CD arm				
**Suspected malaria cases that are managed appropriately (N= ??)**				
CD+PD arm compared to Control arm				
CD+PD arm compared to CD arm				
**Sensitivity 1 – Expanded denominator**				
Consented and met inclusion criteria that are managed appropriately (N=??)				
CD+PD arm compared to Control arm				
CD+PD arm compared to CD arm				
**Untested clients taking ACT**				
CD+PD arm compared to Control arm				
CD+PD arm compared to CD arm				

[8] Minimally adjusted: adjusted only for design variables i.e. strata; fully adjusted: additionally adjusted for pre-specified individual-level and cluster-level variables.

Table B4. Missingness by Treatment Arm

**Variable**[a]	Variable description	**Control (% respondents missing)**	**CD (% respondents missing)**	**CD+PD (% respondents missing)**
test_else	Malaria test elsewhere?			
test_else_res	Result of test elsewhere (among those tested elsewhere, i.e. test_else==1 or test_else == 2)			
test	Did you (or your child) have your blood tested for malaria today at the outlet?			
test_results	RDT results from the outlet (among those tested, i.e. test==1)			
med1	Which medicine(s) did you obtain from the outlet today?			

[a] If there is non-trivial missingness in other variables, we will consider including them in the table as well. We will also consider summarizing characteristics of participants with missing values for these variables if the number of missing values is non-trivial.

Appendix C1: Variables to consider for SES asset index

water	What is the main source of drinking water for your household?
water_sp	Specify other source of water
electricity	Electricity
television	Television
refrigerator	Refrigerator
radio	Radio
phone	Mobile phone (at least one member of the household has)
motorcycle	Motorcycle (at least one member of the household has)
car	Car/Truck
bank	Bank account (at least one member of the household has)
livestock	Does your household have any livestock?
cows	Cows
cows_amt	How many cows does your household have?
sheep	Sheep
sheep_amt	How many sheep does your household have?
goats	Goats
goats_amt	How many goats does your household have?
pigs	Pigs
pigs_amt	How many pigs does your household have?
chickens	Chickens
chickens_amt	How many chickens does your household have?
fuel	What type of fuel does your household mainly use for cooking?
fuel_sp	Specify other fuel used for cooking.
toilet	What kind of toilet does your household have?
toilet_sp	Specify other toilet
house	Do you/your family own the house you live in?
floor	What is the main material of the floor in your house?
floor_sp	Specify other floor material
walls	What is the main material of the walls in your house?
walls_sp	Specify other wall material
roof	What is the main material of the roof of your house?
roof_2	What is the main material of the roof of your house?
roof_sp	Specify other roof material
own_land	Does your household own farmland?
land_size_unit	Unit of measurement for land size
land_acres	Number of acres
land_hectares	Number of hectares
land_sqft_1	Land dimension 1 (shorter side in feet)
land_sqft_2	Land dimension 2 (longer side in ft)
plot	Plot measurement total
school	What is the highest level of schooling you completed?
school_2	What is the highest level of schooling you completed?
occupation	What is your primary occupation?
occupation_sp	Specify other primary occupation
occupation_private_sp	Specify private primary occupation

7 References

1. Hayes RJ, Moulton LH. Cluster Randomised Trials (chapman & Hall/crc Biostatistics Series). 2nd ed. Boca Raton: Chapman And Hall/crc; 2017.
2. Aickin M, Gensler H. Adjusting for multiple testing when reporting research results: the Bonferroni vs Holm methods. Am J Public Health. 1996 May;86(5):726–728.
3. Hemming K, Kasza J, Hooper R, Forbes A, Taljaard M. A tutorial on sample size calculation for multiple-period cluster randomized parallel, cross-over and stepped-wedge trials using the Shiny CRT Calculator. Int J Epidemiol. 2020 Jun 1;49(3):979–995.
4. Zou G. A modified poisson regression approach to prospective studies with binary data. Am J Epidemiol. 2004 Apr 1;159(7):702–706.
5. Zou GY, Donner A. Extension of the modified Poisson regression model to prospective studies with correlated binary data. Stat Methods Med Res. 2013 Dec;22(6):661–670.
6. Zeger SL, Liang KY, Albert PS. Models for longitudinal data: a generalized estimating equation approach. Biometrics. 1988 Dec;44(4):1049–1060.
7. Preisser JS, Young ML, Zaccaro DJ, Wolfson M. An integrated population-averaged approach to the design, analysis and sample size determination of cluster-unit trials. Stat Med. 2003 Apr 30;22(8):1235–1254.
8. Li F, Turner EL, Heagerty PJ, Murray DM, Vollmer WM, DeLong ER. An evaluation of constrained randomization for the design and analysis of group-randomized trials with binary outcomes. Stat Med. 2017 Oct 30;36(24):3791–3806.
9. Prudhomme O'Meara W, Mohanan M, Laktabai J, Lesser A, Platt A, Maffioli E, et al. Assessing the independent and combined effects of subsidies for antimalarials and rapid diagnostic testing on fever management decisions in the retail sector: results from a factorial randomised trial in western Kenya. BMJ Glob Health. 2016 Aug;1(2):e000101.
10. Sullivan Pepe M, Anderson GL. A cautionary note on inference for marginal regression models with longitudinal data and general correlated response data. Communications in Statistics - Simulation and Computation. 1994 Jan;23(4):939–951.
11. Kauermann G, Carroll RJ. A note on the efficiency of sandwich covariance matrix estimation. J Am Stat Assoc. 2001 Dec;96(456):1387–1396.
12. Li P, Redden DT. Small sample performance of bias-corrected sandwich estimators for cluster-randomized trials with binary outcomes. Stat Med. 2015 Jan 30;34(2):281–296.
13. Campbell MK, Piaggio G, Elbourne DR, Altman DG, CONSORT Group. Consort 2010 statement: extension to cluster randomised trials. BMJ. 2012 Sep 4;345:e5661.
14. StataCorp. Stata Statistical Software: Release 16. College Station, TX: StataCorp LLC.; 2019.
15. Preisser JS, Lu B, Qaqish BF. Finite sample adjustments in estimating equations and covariance estimators for intracluster correlations. Stat Med. 2008 Nov 29; 27(27), 5764-5785.
16. Hin LY, Wang YG. Working-correlation-structure identification in generalized estimating equations. Stat Med. 2009; 28: 642–658.
17. Wang X, Turner EL, Li F, Wang R, Moyer J, Cook AJ, Murray DM, Heagerty PJ. Two weights make a wrong: Cluster randomized trials with variable cluster sizes and heterogeneous treatment effects. Contemp Clin Trials. 2022 Mar;114:106702.
18. Kahan BC, Li F, Copas AJ, Harhay MO. Estimands in cluster-randomized trials: choosing analyses that answer the right question. Int J Epidemiol. 2022 Jul 14:dyac131.
19. Rutsein, S. O. & Johnson, K. The DHS Wealth Index. (Calverton, Maryland, USA, 2004).

20. Kolenikov, S. & Angeles, G. Socioeconomic Status Measurement with Discrete Proxy Variables: Is Principal Component Analysis a Reliable Answer? *Review of Income and Wealth* **55**, 128-165, doi:10.1111/j.1475-4991.2008.00309.x (2009).
21. Kolenikov, S. & Ángeles, G. The use of discrete data in principal component analysis with applications to socio-economic indices. CPC. (MEASURE Working paper no. WP-04-85, 2004).
