# Supplementary material for: A cluster-randomized trial of client and provider-directed financial interventions to align incentives with appropriate case management in retail medicine outlets: Results of the TESTsmART Trial in western Kenya
Source: PLOS Glob Public Health. 2024 Feb 7;4(2):e0002451. doi: 10.1371/journal.pgph.0002451 (PMC10849268; doi:10.1371/journal.pgph.0002451)
Supplement: S2 Text — (PDF) [file pgph.0002451.s005.pdf]

A randomized experiment of malaria diagnostic **te**sting and conditional **s**ubsidies to **t**arget **A**CTs in the **ret**ail sector: the TESTsmART trial AIM 2

Sub-Title: Outlet-based trial of RDTs and conditional ACT subsidies among patients with fever

DMID Protocol Number:

Sponsored by:
National Institute of Allergy and Infectious Diseases (NIAID)

Principal Investigators:
Wendy Prudhomme O'Meara
Theodoor Visser
Jeremiah Laktabai

Draft Version Number: *1.5*

Day Month Year
1 Sept 2022

Statement of Compliance

The study will be carried out in accordance with the principles set forth in The Council for International Organizations of Medical Sciences (CIOMS) International Ethical Guidelines for Biomedical Research Involving Human Subjects and 45 CFR Part 46.

All key personnel (all individuals responsible for the design and conduct of this study) have completed Human Subjects Protection Training.

SIGNATURE PAGE

The signature below constitutes the approval of this protocol and the attachments, and provides the necessary assurances that this trial will be conducted according to all stipulations of the protocol, including all statements regarding confidentiality, and according to local legal and regulatory requirements and applicable US federal regulations and ICH guidelines.

Site Investigator:*

Signed: ______________________________ Date: ______________

Wendy Prudhomme-O'Meara, PhD
Principal Investigator

** The protocol should be signed by the local investigator who is responsible for the study implementation at his/her specific site; ie, if Investigational New Drug study, the individual who signs the Form FDA 1572.*

Table of Contents

page
Statement of Compliance i
Signature Page ii
List of Abbreviations iv
Protocol Summary v

1 Background Information and Scientific Rationale 5
1.1 Background Information 5
1.2 Scientific Rationale 7
1.3 Potential Risks and Benefits 8
1.3.1 Potential Risks 8
1.3.2 Known Potential Benefits 8
2 Objectives 10
3 Study Design 11
4 Study Population 14
4.1 Selection of the Study Population 14
4.2 Inclusion/Exclusion Criteria 14
5 STUDY PROCEDURES/EVALUATIONS 16
5.1 Study Procedures 16
5.2 Laboratory Evaluations 17
6 Statistical Considerations 18
6.1 Study Outcome Measures 18
6.2 Sample Size Considerations 19
6.3 Participant Enrollment and Follow-Up 20
6.4 Analysis Plan 21
7 SUBJECT CONFIDENTIALITY 23
7.1 Future Use of Stored Specimens 23
8 Informed Consent Process 24
8.1 Informed Consent/Assent Process (in Case of a Minor or Others Unable to Consent for Themselves) 24
9 RESULT DISSEMINATION PLAN 26
10 Literature References 27

SUPPLEMENTS/APPENDICES
1. Exit Interview Questionnaire
2. Adult Verbal Consent Form (attached separately)
3. Outlet Flyer (attached separately)
4. Outlet enrollment questionnaire
5. Outlet consent form

ACT	Artemisinin Combination Therapy
AE	Adverse Event
AM	Antimalarial
CFR	Code of Federal Regulations
CIOMS	Council for International Organizations of Medical Sciences
CRF	Case Report Form
CHW	Community Health Worker
DFID	Department for International Development, UK
DMID	Division of Microbiology and Infectious Diseases, NIAID, NIH, DHHS
DSMB	Data and Safety Monitoring Board
FWA	Federal-Wide Assurance
GCP	Good Clinical Practice
GEE	Generalized Estimating Equation
ICF	Informed Consent Form
ICH	International Conference on Harmonisation
ID	Identification
IEC	Independent or Institutional Ethics Committee
IRB	Institutional Review Board
ISM	Independent Safety Monitor
JAMA	Journal of the American Medical Association
MOP	Manual of Procedures
N	Number (typically refers to subjects)
NEJM	New England Journal of Medicine
NIAID	National Institute of Allergy and Infectious Diseases, NIH, DHHS
NIH	National Institutes of Health
OCRA	Office of Clinical Research Affairs, DMID, NIAID, NIH, DHHS
OHRP	Office for Human Research Protections
ORA	Office of Regulatory Affairs, DMID, NIAID, NIH, DHHS
PI	Principal Investigator
PPMV	Patent and Proprietary Medicine Vendors, Nigeria
RA	Research Assistant
RD	Risk Difference
RDT	Rapid Diagnostic Test
RR	Risk Ratio
SAE	Serious Adverse Event
SMC	Safety Monitoring Committee
SOP	Standard Operating Procedure
USAID	United States Agency for International Development
WHO	World Health Organization

Title: A randomized experiment of malaria diagnostic **te**sting and conditional **s**ubsidies to **t**arget **A**CTs in the **ret**ail sector: the TESTsmART trial AIM 2

Population: 40 registered pharmacies in western Kenya, 6800 participants seeking care from these pharmacies

Number of Sites: 1

Study Duration: 24 months

Subject Duration: 30 minutes

Objectives:
The ultimate goal of a conditional subsidy scheme is to limit inappropriate use of ACTs and reduce the programmatic costs of ACT subsidies by offering them conditional upon a positive test at the point of sale.

The objective of this study is to test the effect of provider-directed and patient-directed incentives on improving the management of suspected malaria fevers that receive care in the retail sector. Provider-directed incentives include small payments for taking the time to conduct malaria-RDT testing for participants with malaria-like illness. Patient-directed incentives are inexpensive RDT testing coupled with a conditional ACT discount. The ACT discount is only applied if the RDT is positive for malaria. Outcomes will be measured by exit interviews on random days each month at each participating outlet.

Primary:

- The primary outcome will be the proportion of all ACTs that are sold to individuals with a positive malaria diagnostic test. For this outcome, a positive test is anyone who has a malaria-RDT performed at the outlet and receives a positive result or is referred from a health facility with a documented positive test result.

Secondary:

- The major secondary outcome is the proportion of suspected malaria cases that are tested. This outcome will allow us to determine whether the conditional subsidy can drive demand for testing.
- Other secondary outcomes will measure 1) adherence to the RDT result among all those tested at the outlet, 2) proportion of all suspected malaria cases that are managed appropriately (tested for malaria, and use ACT following a positive test or do not purchase an ACT after a negative test), and 3) ACT use by untested clients.

For questions regarding this protocol, contact:

Individuals: **DMID Representative:**
Walt Jones, RN, MPH
Clinical Project Manager NIH/NIAID/DMID/PIPB
5601 Fisher's Lane, Room 8A37, Rockville, MD 20892
PHONE: 240-627-3283
joneswalter@niaid.nih.gov

Principal Investigator: *Site investigator responsible for conducting the study:*
Wendy Prudhomme O'Meara, PhD
Associate Professor of Medicine and Global Health, Duke University
Box 90519, Trent Hall, Durham, NC 27708
PHONE: +254 728 306 521
wpo@duke.edu

Subcontract Principal Investigators:
Theodoor Visser
Senior Manager, Malaria Commodity Access, Clinton Health Access Initiative
383 Dorchester Ave., Ste 400, Boston, MA 02127
FAX: 617 774 0220
PHONE: 706 296 4141
tvisser@clintonhealthaccess.org

Jeremiah Laktabai, PhD
Lecturer, Department of Medicine, Moi University School of Medicine
P.O. Box 4605-30100 MTRH Complex, Nandi Road, Eldoret, Kenya
FAX: +254 532 061 992
PHONE: +254 722 685 542
jlaktabai@gmail.com

Institutions:
Duke University
Box 90519, Room 219, Trent Hall Durham, NC 27708
Jamie Mills, Lead Grants and Contracts Administrator, DGHI
FAX: 1 919 681 7748
PHONE: 1 919 684 1755
jamie.mills@duke.edu

Clinton Health Access Initiative (CHAI)
383 Dorchester Ave., Ste 400, Boston, MA 02127
Molly Baker, Finance and Operations Manager, Access Programs, CHAI
FAX: 617 774 0220

PHONE: 617 774 0110
mbaker@clintonHealthAccess.org

Moi University
PO Box 4606-30100 MEC Building, Nandi Road, Eldoret, Kenya
Robert Rono, Head of Research and Sponsored Projects Office
FAX: +254 532 061 992
robertrono@iukenya.org

1 BACKGROUND INFORMATION AND SCIENTIFIC RATIONALE

1.1 Background Information

Artemisinin combination therapies (ACTs) – the WHO-recommended first line therapy for uncomplicated malaria – have played a significant role in reducing global malaria mortality [1], but their overuse is rampant. In 2016 an estimated **216 million cases of malaria occurred worldwide, yet more than 400 million treatment courses of ACT were consumed**[1] [2]. Approximately 75% of global ACT demand is subsidized with international public funds from sources such as The Global Fund, DFID, and USAID[2] [3] . Overconsumption of ACTs is an unnecessary drain on scarce public health resources and threatens the future sustainability of publicly-funded subsidies. In addition, it puts both present and future patients at risk; inappropriate treatment of a non-malaria illness with an antimalarial increases case fatality rates [4, 5] and contributes to population-wide drug pressure that accelerates the spread of drug resistance [6-8].

Global over-consumption of ACTs is largely driven by its increased over-the-counter distribution in private retail outlets as a result of publicly-funded subsidies directed to the private sector [9]. In 2015, **44% of all donor-funded ACTs consumed world-wide were distributed through the private retail sector** [9] where studies have shown that between **65-91% of ACTs dispensed for malaria are actually purchased by people without malaria** [10-13]. Targeting ACTs to only those who receive a confirmatory diagnosis could dramatically reduce inappropriate ACT consumption, in alignment with WHO policy that stipulates that all febrile patients be tested before administering antimalarials.

Although more than half of families in sub-Saharan Africa seek care for febrile illness through the retail sector [14, 15], it has remained largely outside of efforts to improve rational, or diagnosis-directed, use of antimalarials. Private medicine retail outlets are made up of for-profit outlets that specialize in medicines, such as pharmacies and drug stores. These outlets are generally poorly regulated, often operate outside of formal channels, and have weak, sometimes antagonistic relationships with the formal health care sector. The lack of accountability and incentive structure undermines adherence to national case management guidelines and contributes to poor ACT stewardship in the retail sector.

Point-of-care malaria rapid diagnostic tests (RDTs), which have excellent sensitivity and specificity and are simple enough to be used by trained laypersons with limited formal training [16], could expand the reach of diagnostics into the retail sector and help improve the rational

[1] This includes both 311 million courses of subsidized, WHO-prequalified ACTs as well as an estimated figure of more than 100 million courses of other brands not subsidized or tracked by the WHO. Quality-assured or pre-qualified ACTs are pre-approved ACT brands that meet WHO quality standards. Only quality-assured ACTs are subsidized.
[2] USAID – United States Agency for International Development; DFID – Department for International Development, UK

use of antimalarials. However, in the context of subsidized ACTs and a for-profit business model, it may not be in the economic interest of clients to test when the treatment is less expensive than the RDT. Clients may prefer to forgo the RDT in favor of the treatment if they strongly suspect they have malaria, particularly if testing may increase their out-of-pocket costs. Similarly, use of a test may also mean loss of a sale for the retailer if the test is negative, particularly if there is an expectation that the provider will refer all clients who test negative for further evaluation at a formal health facility, as has been suggested by policy-makers. Therefore, in the presence of highly subsidized ACTs, incentives for testing are not aligned with provider or client interests in the private retail sector. **As a result, the private retail sector remains the largest contributor to inappropriate use of ACTs.**

Currently, recipients of Global Fund grants may use funds to subsidize RDTs in the private sector, including in private retail outlets. However, outside of a few countries (i.e., Cambodia, Ghana, Myanmar), the use of RDTs in the retail sector is uncommon as is evidenced by the very low testing rates (<10%) in the private sector in sub-Saharan Africa [2, 17]. Several studies have explored the potential role of RDTs in improving case management in the retail sector with mixed and often poor results. In most of these studies retail providers received case management training, followed by supportive supervision visits by researchers. In a few studies, the wholesale RDT price was partially or fully subsidized but retail providers were permitted to set their own price to the consumer and offer testing at their discretion [18, 19]. More often, outlets were required to provide testing free of charge or at a low fixed price. Instructions to the outlets regarding when an RDT should be performed and an ACT should be dispensed were quite rigid [20-23]. Providers were not explicitly incentivized to conduct RDTs. All of these studies shared two features – 1) ACTs were heavily subsidized for all customers and 2) there was no relationship between the RDT result and the ACT subsidy. This range of implementation strategies resulted in a wide range of testing uptake; between 7 - 100% of suspected malaria cases were tested [24]. Adherence to a negative malaria test was inconsistent (between 1-40% of those with a negative test purchased an ACT) and often a significant portion of those testing positive, up to 70%, did not take an ACT [24].

In order to align both the provider and customer incentives towards testing and targeting, the provider must be willing to perform the test and sell the appropriate medicine and, at the same time, the customer must be motivated to purchase the test and adhere to the results. We hypothesize that offering ACT subsidies for the client (conditional on a positive test), will have a very modest impact on uptake of testing and targeting of ACTs in the retail sector. We further hypothesize that when combined with a provider-incentive to offer malaria testing, they will have a synergistic effect on RDT testing and ACT targeting. Although it would be beneficial to estimate the effect of each intervention separately as well as in combination in order to provide more comprehensive evidence to support joint implementation, preliminary data from the study area indicates that the study may not be powered to measure the effect of a four-arm study. We believe that the conditional ACT arm and combination RDT provider incentive and conditional ACT arm

comparison will sufficiently estimate the incremental impact of the RDT provider incentive. Additionally, provider-directed incentives alone may not be as impactful for policymakers. We will test the combination of subsidies for ACTs and RDTs (selected from Aim 1) paired with a provider-directed testing incentive in a three-arm cluster-randomized controlled trial to evaluate their impact on the proportion of ACTs sold to individuals with parasitologically-confirmed malaria among those seeking care in the retail sector.

1.2 Scientific Rationale

The study will be conducted in western Kenya, where the retail sector is an important source of malaria treatment. 100% of the population is considered at risk for malaria, and the private sector is an immensely important avenue for fever management. Nationwide surveys show that 70.6% of ACTs are distributed through the private sector [25]. Given the large proportion of individuals obtaining anti-malarial drugs outside the formal sector, efforts to improve targeting of ACTs must include the retail sector.

Kenya has the programmatic goal of universal access to prompt parasitological diagnosis before treatment and has endorsed the use of malaria RDTs in the public health sector. However, very high rates of self-treatment in the retail sector undermine the explicit policy that all malaria cases should be confirmed by parasitological diagnosis. Private medicine retailers in Kenya, consisting of private pharmacies and chemists, are not routinely permitted to conduct RDTs. Instead, emphasis has been placed on shifting care to the formal health sector or deploying RDTs in the community through lay health workers. Persistently low testing rates have led Kenya to recently reconsider its position on RDT use in the retail sector, but the country has delayed any changes due to lack of rigorous data to guide policy.

Table 1: Study country characteristics

	**Kenya**
First line drug	AL[1]
Year adopted universal testing policy	2010
Estimated malaria cases (2016) [26]	3.5 million
Total population	50M
Percent of cases confirmed [26]	33%
Percent of fevers that seek care in retail sector	60%
Current price of ACT in retail sector (adult dose)	$1.20
Expected price in 2018	$1.20
Use of RDTs in retail sector?	No
Current retail cost of RDT	$1.00 [27]
Study area	Rural
Prevalence of malaria in study area	25%

[1] [illegible]

While there exist simulations of clients' decisions around testing and ACT purchasing based on prices of these commodities [25], they do not consider the motivations of providers in the retail sector to offer malaria diagnostic testing. The proposed project is a real-world implementation study that will provide insights into whether an innovative and scalable approach can help overcome the practical challenges of working through the private retail sector while improving the targeting of ACTs to individuals with confirmed malaria infection.

1.3 Potential Risks and Benefits

1.3.1 Potential Risks

The intervention proposed is an incentive to providers to perform malaria diagnostic testing and the offer of a conditionally subsidized ACT for the client in case of a positive test. The provider incentive is relatively small, so it is not likely to distort the providers' behavior to the extent where they would perform unnecessary malaria tests in order to obtain the incentive. The subsidized ACT is offered only to patients with a positive test (and only in some arms) but, the client is free to choose whether or not to use the subsidy. Participating in this study involves allowing us to record information about clients' malaria testing and treatment decisions. This includes the clients' decisions about whether to get tested for malaria using the RDT, and whether to buy an ACT. There is a small risk of breach of confidentiality of this information.

RDTs will be provided to all participating pharmacies for wholesale price. The RDTs to be used in this study are the same brand and test as those used by the Government of Kenya in public health facilities and in their community-based case management for malaria. The study will ensure availability of proper sharps disposal equipment and services to participating outlets. All clients who choose to be tested will be advised on what to do if they experience any adverse events as a result of the finger prick.

Under our Quality Assurance Exit Testing activities, we will ask a subsample of those who participate in the exit interviews if they are willing to be tested for malaria with an RDT as they leave the outlet. Some of those participants will already have been tested in the outlet. This exit test will allow us to 1) determine whether shops are providing correct diagnosis and 2) measure what proportion of untested clients are not receiving appropriate management for their undiagnosed malaria illness. A finger prick blood sample is required to test for malaria and poses a small risk of temporary discomfort and, very rarely, infection at the site of the prick.

Interviews and focus group discussions are designed to elicit opinions and perspectives of participants and are a minimal risk activity. Participation does pose a small risk of breach of confidentiality if the identities of the participants become known. There is also a risk of psychological discomfort if other participants react negatively to some expressed views.

1.3.2 Known Potential Benefits

There is significant health benefit to the client in knowing their malaria infection status prior to purchasing a drug. There is also a benefit to the client to be able to purchase an effective drug at a reduced, fixed price when they have a confirmed malaria infection, which may also reduce the likelihood that they would purchase an inappropriate or outdated therapy.

In one arm, the providers get a direct monetary incentive for performing malaria diagnostic tests.

For those tested as part of the Quality Assurance Exit Testing, there is benefit to ensuring the test results are correct or to knowing their malaria infection status if they chose not to pay for a test in the shop. In addition, participants who test positive for malaria at the exit test who did not receive AL will have the opportunity to get AL to treat their malaria free of charge.

More broadly, there are important future benefits to rigorous testing of subsidy schemes that promote appropriate testing before treatment. This work will contribute to evidence-based policy making, improved access to malaria diagnosis and ultimately reduced potential for the spread of antimalarial resistance.

2 OBJECTIVES

The ultimate goal of a conditional subsidy scheme is to limit both inappropriate use of ACTs by those without a test or with a negative test, as well as to reduce the programmatic cost of ACT subsidies by offering them conditionally on a positive test at the point of sale. The objective of this study is to test the effect of provider-directed and patient-directed incentives on improving the management of suspected malaria fevers that seek care in the retail sector.

3 STUDY DESIGN

This will be a three-arm cluster-randomized trial reduced from an underlying 2x2 factorial design. A random sample of 40 pharmacies (clusters) will be selected from a complete sampling frame of all eligible outlets and subsequently randomly assigned in a 14:13:13 ratio to each of the 3 arms (see Table 2).

All clusters (Arm 1-3) will have access to RDTs at the wholesale price that enables the outlet to charge the desired retail price. The study will pre-specify the retail price so that it is consistent across all the arms and pharmacies and will work with in-country wholesalers to provide RDTs at the appropriate price to all participating outlets. In addition, all outlets will be trained on a mobile reporting app and asked to use it to record RDT and ACT sales and to facilitate payment of financial incentives. The three treatment arms are as follows:

(1) Control: No price subsidy or incentive. RDTs are made available at wholesale price to the retail outlet.

(2) Client-directed conditional ACT subsidy (CD): Clients visiting outlets in this arm will receive a free ACT *if* they purchase a malaria test and have a positive test result.

(3) Combined interventions (PD-CD): Retail outlets in this arm receive an incentive to test for malaria and clients visiting these outlets receive a free ACT conditional on a malaria positive test (i.e. this arm is a combination of the provider-directed and client-directed interventions).

Our three-arm study design will allow us to measure the effect of joint incentives to the provider and consumer, relative to no incentives and relative to the client-directed incentive alone.

Data will be collected by two independent mechanisms – provider reporting and exit interviews. First, we will examine routine reporting data submitted via mobile phones using the mobile app. All shopkeepers within each enrolled outlet will be trained to use the mobile app which reports on volume of clients, number of ACTs or other antimalarials sold, number of RDT sold. The mobile reporting app will also require the user to take a picture of every RDT test. These photos will be uploaded to the server in real-time and will be reviewed continuously by registered lab techs. Data reported through the app will primarily be used to track RDT and ACT sales in real-time and will be regularly reviewed to track proportion of positive tests, volume of RDTs used, and visualization of a random sample of uploaded RDT photos. This routine monitoring will detect potential problems (i.e. providers who have unusually high or low test positivity rate, problems with RDT interpretation). Problems detected will trigger support supervision and/or additional on-the-job training to ensure compliance and quality of diagnosis.

Data for our main study outcomes will be collected by exit interviews with customers in order to avoid bias that may arise by relying on provider-reported data. In previous retail-sector studies, results from exit interviews and provider reports differed significantly, with exit interview results considered more reliable [22, 26]. Trained data collectors will approach customers who have transacted at the outlet and ask them if they are willing to participate in an exit interview. In our experience, refusal rates are less than 2% for these types of surveys so we expect our sample to accurately represent all transactions at the outlet. Customers will be eligible if they sought treatment for a febrile illness or malaria-like symptoms for themselves or their child, provided the child is present. Exit interviews will be conducted on randomly selected days each month and data collectors will be randomly assigned to outlets in order to minimize behavior change prompted by the presence of the interviewer. The number of days of data collection at each outlet will depend on the sales volume with a target of 10 participants per outlet per month. Customers will be asked to report whether they had a test, the results of the test, and what medicine they purchased. Basic information about household assets will also be collected in order to stratify participants on socioeconomic status.

Exit interview and provider reporting data will be compared to assess agreement between the sources. Specifically, we will compare the following indicators aggregated by outlet: the proportion tested, the proportion of tests positive and, the proportion of individuals using ACTs by test status (untested, positive or negative).

The primary outcome will be the proportion of all ACTs that are sold to individuals with a positive malaria diagnostic test. The ultimate goal of a conditional subsidy scheme is to limit both inappropriate use of ACTs by those without a test or with a negative test as well as to reduce the programmatic cost of ACT subsidies by offering them conditionally on a positive test at the point of sale. For this outcome, a positive test is anyone who is tested at the outlet and receives a positive result or is referred from a health facility with a documented positive test result. Although all ACTs purchased after a test, whether tested at the outlet or health facility, are included in the outcome measure, only ACTs purchased following *a test at the outlet* are eligible for the conditional subsidy. Our major secondary outcome is proportion of suspected malaria cases that are tested. This outcome will allow us to determine whether the conditional subsidy can drive demand for testing. Other secondary outcomes will measure 1) adherence to the RDT result among all those tested, 2) proportion of all suspected malaria cases that are managed appropriately (tested for malaria, and use ACT following a positive test and do not purchase an ACT after a negative test), and 3) ACT use by untested clients.

- Study outcomes will be supplemented with qualitative data collected during focus group discussions and key informant (in depth) interviews. The goal of the qualitative work is threefold –

1) To understand the perspectives of outlets that interacted with the intervention regarding malaria diagnosis, the effectiveness of the intervention, and the feasibility or value of scaling up or modifying the intervention

2) To understand the perspectives of outlets naïve to the intervention regarding malaria diagnosis, the risks and benefits of presumptive malaria treatment and how outlets could or should be engaged in Kenya’s Test before Treat policy
3) To learn from leadership their perspectives on how the services provided in the private retail sector can contribute to the Kenyan governments plans for Universal Health Care, including fever management

This information will help place quantitative results into context, provide insight into heterogenous intervention effects, and help translate findings into recommendations for implementation in a more robust manner.

4 Study Population

4.1 Selection of the Study Population

All clients attending a participating outlet on the day selected for exit interviews will be eligible to be screened for inclusion into the interview sample. Only those who meet all the criteria will be counted towards the sample size.

The first two exit interview participants at each outlet on a given data collection day will be asked to participate in additional Quality Assurance Exit Testing.

At the conclusion of the trial, focus group discussions will be conducted with outlets selected from the sampling frame stratified by participation in the study (i.e. outlets who participated in the study and outlets which were eligible but were not sampled or randomized to the study). We will also conduct a focus group discussion with data collectors to provide an opportunity for them to share observations from their interviews with clients that were not captured in the survey tool. We will also conduct in-depth interviews with two community health workers, three outlet owners, and two local ministry of health officials.

4.2 Inclusion/Exclusion Criteria

OUTLETS: Only outlets who are registered with the Kenya Pharmacy and Poisons Board and who stock ACTs will be asked to participate

PARTICIPANTS-

INCLUSION CRITERIA:

- Participants with fever, or history of fever in the last 48 hours, or suspects they may have malaria
- Individual with malaria-like illness must be present at recruitment
- Older than one year of age

EXCLUSION CRITERIA:

- Any individual with signs of severe illness requiring immediate referral
- Individuals who have taken an antimalarial in the last seven days, including for the current illness
- Patients <18 years without a parent or legal guardian present
- Adults who are unable to consent for themselves

5 STUDY PROCEDURES/EVALUATIONS

5.1 Study Procedures

On random days of the month, clients leaving the pharmacy will be asked to participate in a brief survey. Those who meet the inclusion/exclusion criteria will provide verbal consent before responding to questions about their current illness and their decisions regarding testing and medicines purchased. These clients will have been offered an RDT at the pharmacy if they have malaria-like symptoms. RDTs will be available in all participating pharmacies at the same price. Those who do not wish to purchase an RDT are free to conduct their transaction as planned.

The exit survey will be conducted in one session and last approximately 15-20 minutes.

Quality Assurance Exit Testing: A subset of those who participate in the exit interview will be asked if they are willing to take an mRDT offered free of charge, conducted by our study team. Written informed consent will be collected from participants who agree to join the QAET subsample. These participants will undergo an mRDT conducted by our trained research staff, free of charge. The results of the QA test will be recorded along with the participants study ID. In most cases, the participant may not want to wait for the results of the test. Therefore, a card with the study ID and the study phone number will be given to each participant. A small amount of airtime (200 KES) will be provided to each participant to allow them to call the study phone to learn their results. If their results show that they have malaria, we will provide the appropriate dose of first-line antimalarial to anyone who did not get one at the shop.

Focus group discussions: Participants will be invited to meet at a centralized location on a specific day. The purpose and content of the discussion will be explained after which they will be asked to provide informed consent for participation and recording of the discussion. Respect for others' views and opinions and confidentiality will be stressed to the participants. A focus group moderator will guide the discussion using the tool provided. A note-taker will also be present. Both the moderator and notetaker will be external to the study implementation team in order to avoid bias arising from prior working relationships, knowledge of the study, or investment in the study outcome. Discussions will last 60-90 minutes.

We will conduct 5 FGDs with 6-8 participants each. We will convene two FGDs with outlets in the intervention arms, stratified by geographic location, and one FGD with outlets in the comparison arm. Focus group discussion with outlets not involved with the study – we will convene two FGDs with 6-8 participants who are attendants or owners of registered outlets nearby the study area but not part of the study. Focus group discussions with data collectors – data collectors regularly spent time at every participating shop for over a year. Their observations and feedback could offer important insights beyond the statistics that were captured in the exit interviews. We will have one FGD with 6-8 exit interviewers.

a. See Verbal consent for FGD participation (Appendix X)
b. See FGD discussion guide for study outlets (Appendix X)
c. See Verbal consent for FGD participation (Appendix X)
d. See FGD discussion guide for non-intervention outlets (Appendix X)
e. See Verbal Consent for FGD participants (Appendix X)
f. See FGD discussion guide for data collectors (Appendix X)

In-depth interviews: One-on-one conversations with key informants will be scheduled in advance and be carried out in a private location. After explaining the purpose and content of the interview, consent will be obtained for participation and recording of the conversation. Interviews will last no more than one hour. In depth interviews will complement the FGD data by providing perspectives of health leadership and community leaders. In addition, we will conduct IDIs with several outlet owners to triangulate information from the FGDs and ensure that our FGDs were comprehensive enough to cover all the major themes. IDI participants will include: County or sub-County Malaria Control Coordinator and Clinical Officer In-Charge from Bungoma and Trans Nzoia (or relevant subcounties within); Community Health Workers; two outlet owners from intervention outlets; one outlet owner from non-participating outlet.

5.2 Laboratory Evaluations

None

6 STATISTICAL CONSIDERATIONS

6.1 Study Outcome Measures

The primary outcome measure is the proportion of all ACTs that are sold to individuals with a positive malaria diagnostic test defined as anyone who is tested at the outlet and receives a positive test result *or* is referred from a health facility with a documented positive test result**.**

Among secondary outcomes, behavior will be measured for individuals who were tested at the outlet. Untested clients are those who chose not to be tested and did not come with a test result from another facility. The major secondary outcome is the proportion of suspected malaria cases that are tested. This outcome will allow us to determine whether the conditional subsidy can drive demand for testing. Note that “untested people” here refers specifically to individuals that present at the shop without a referral but decline to be tested

$$\frac{\#\,people\ who\ were\ tested\ with\ an\ RDT}{\#\,people\ who\ were\ tested\ with\ an\ RDT + \#\,untested\ people\ who\ purchased\ an\ antimalarial}$$

Other secondary outcomes will measure 1) adherence to the RDT result among all those tested at the outlet, 2) proportion of all suspected malaria cases that are managed appropriately (tested for malaria, and use ACT following a positive test or do not purchase an ACT after a negative test), and 3) ACT use by untested clients.

- Secondary outcome 2: Adherence to the RDT result among all those tested in the shop

$$\frac{\#\,people\ who\ tested\ positive\ and\ purchased\ ACT + \#\,people\ tested\ negative\ and\ did\ not\ purchase\ any\ AM}{\#\,people\ who\ were\ tested\ with\ an\ RDT}$$

For those who are negative, if they buy any antimalarial (AM), including monotherapies and older therapies, they are not adhering to test result.

- Secondary outcome 3: Proportion of all suspected malaria cases that are managed appropriately in the shop

$$\frac{\#\,people\ who\ tested\ positive\ and\ purchased\ ACT + \#\,people\ tested\ negative\ and\ did\ not\ purchase\ any\ AM}{\#\,people\ who\ were\ tested\ with\ an\ RDT + \#\,untested\ people\ who\ purchased\ an\ antimalarial}$$

“Untested people” here refers specifically to individuals that present at the shop without a referral but decline to be tested

- Secondary outcome 4: ACT use among untested clients

$$\frac{\#\,people\ who\ were\ not\ tested\ in\ the\ shop\ and\ purchased\ an\ ACT}{\#\,people\ who\ were\ not\ tested\ in\ the\ shop, did\ not\ have\ documentation\ of\ test\ and\ purchased\ any\ AM}$$

For the primary outcome, we include in both numerator and denominator those individuals who were referred from a health facility with a documented positive test result. That is, for the

primary outcome we are interested in evaluating the degree to which the interventions impacted the purchasing behavior of all suspected malaria cases seeking treatment. Our secondary outcomes do *not* include these individuals (i.e. the outcomes are calculated on the subset of individuals that present at the shop for testing without a referral). That is, for the secondary outcomes we are specifically interested in evaluating the degree to which the interventions impacted purchasing behavior among individuals who present at the shop for testing.

6.2 Sample Size Considerations

Our primary comparison of interest is the effect on targeting of ACTs of offering a combination of outlet incentives for testing (provider-directed intervention), and ACT subsidies for malaria test-positive clients (client-directed intervention) relative to a control arm where outlets are able to offer malaria testing but neither the provider nor the client receives any extrinsic incentive to test (Arm 3 versus Arm 1). We also have a secondary comparison of interest: the combined provider- and client-directed interventions relative to the client-directed intervention alone (Arm 3 versus Arm 2).

We calculated power based on a cluster randomized two-sample two-tailed t-test for the comparison of two proportions using standard formulae [27]. We assumed 39 clusters (outlets) would be available to be enrolled based on information about the number of registered pharmacies in the study area. In practice we will have an extra outlet to be assigned to the control arm, so the power calculation is conservative. We calculated power for differences in our primary outcome for each of the two comparisons of interest noted above. To ensure that our overall two-tailed Type I error (alpha) is 5%, we fixed the alpha level at 2.5% (i.e. 5%/2) for each of the 2 comparisons of interest, using the conservative Bonferroni correction [28]. We estimated the intra-class correlation coefficients (ICCs) for the primary outcome to be 0.009 in Kenya.

Table 2: Assumptions in Kenya and Nigeria for sample size calculation for Aim 2

	**Expected Percentage of ACTs taken by Clients Testing Positive for Malaria**	**Power (# of exit interviews per arm)**
	Kenya	Kenya (2210)
**Arm 1: Control Arm** (RDT only, no incentives)	7%	99.1%
**Arm 2: Provider-Directed Intervention** (Shopkeeper incentives for malaria testing)	NA	NA
**Arm 3: Client-Directed Intervention** (ACT subsidy to client conditional on positive test)	12%	80.2%

Our primary outcome—the proportion of ACTs that are taken by malaria test-positive clients— is a combination of the proportion of individuals who get tested for malaria and the proportion of tested individuals who treat according to the test result. We anticipate that 7% of ACTs will be purchased by clients with a positive test in the control arm (with 93% of ACTs purchased by those without a test or with a negative test). Moreover, we hypothesize that the client directed intervention on its own will only increase testing marginally (on the order of ~5 percentage points), but that among those who do test, the client-directed intervention will substantially increase the proportion of malaria-positive individuals who take an ACT [29]. As a result, we expect that the client-directed intervention (Arm 2) will have a somewhat large effect, but that a larger effect will come from combining the two interventions (Arm 3) (i.e., we assume a statistical interaction). With a sample of 170 exit interviews per outlet (40 outlets in Kenya), we will have >90% power to detect a minimum difference between Arms 1 and 3 in the primary outcome of 16 percentage points. We will also have >80% power to detect a minimum difference of 11 percentage points for the main secondary comparison of interest (testing uptake).

Due to the nature of the interventions, it is not possible to blind participants and the implementation team to the allocation received. Data collectors will be blinded throughout collection and study statisticians will be blinded during the analysis phase.

During the last two months of data collection, we will enroll an estimated 300 exit-interview participants in exit testing for quality assurance. We will compare the results from outlet testing and results from the study provided-RDT using a kappa statistic. Given the assumed value of kappa of 0.26 and the standard deviation is 1.32, a sample size of 300 subjects results in a two-sided 95% confidence interval of 0.11 to 0.41, with a width of 0.30.

6.3 Participant Enrollment and Follow-Up

Medicine retail outlets will be eligible if they regularly stock and sell regulatory approved artemether-lumefantrine (AL) and are licensed medicine outlets. From this roster of all eligible

outlets, 41 will be randomly selected (2 additional above the required sample size). Of those retail outlets, 40 outlets will be randomly selected for training and enrollment in the study and will be randomized to Arms 1-3 in a 14:13:13 ratio. The remaining outlet will be an alternate in the event that any of the initial outlets drop out of the study. The alternate outlet will be allocated as control (they will receive RDTs at wholesale price and will be trained on the app), but will not undergo arm specific training unless it is needed for replacement in either intervention arm.

We will retain these enrolled outlets through effective supportive supervision and communication between providers at participating outlets and study staff. All outlets will receive thorough, in-person training on using the mobile reporting tool, conducting RDTs and overall study procedures. They will also receive extra support during the start of the intervention and burn in period (3 months), including an initial stock of RDTs provided by the study at no cost. At least once per month, the PI, Project Manager, and/or Field Coordinator will visit each outlet in person to answer questions, check RDT stock and testing performance, and identify and address any problems. Providers will also receive phone calls from the Field Coordinator and Project Manager between in-person visits and will themselves be provided with phone numbers and encouraged to call the study staff with any questions or concerns. Additionally, outlets will be reimbursed promptly to ensure participation in the study presents no financial strain.

In our previous work, we have had excellent retention of outlets in research activities and therefore have no reason to expect attrition of clusters. However, should one of the outlets selected choose to leave the study within the first 6 months of the intervention, the outlet will be replaced by one of the alternate outlets assigned to the same arm. If any outlet chooses to leave the study after the first 6 months of the intervention, they will not be replaced because, after joining the intervention at such a late stage, a newly added outlet is not likely to be comparable to that of all other participating outlets. Changes in the clusters (dropped and/or replaced) will be accounted for in the analysis.

Our sample size estimates correspond to a total of 6800 exit interviews with clients. Since not everyone interviewed will have purchased an ACT, our estimates account for the fact that only a subset will enter into our analysis for the primary outcome.

6.4 Analysis Plan

We will analyze client-level outcomes by fitting a modified Poisson regression model [30, 31] with log link to estimate risk ratios (RRs) and identity link to estimate risk differences. Such an approach assumes a Poisson distribution for the binary outcome and then ‘fixes’ the estimated standard errors to correct for model misspecification.

To account for clustering by outlet we will use a generalized estimating equations (GEE) [32, 33] approach with exchangeable working covariance matrix and robust standard errors (to correct for model misspecification due to specifying a Poisson distribution). The outcome will be regressed on two binary indicators for each of the treatment arms 1 and 2, with treatment arm 3 (the combined interventions) serving as the reference group. The model will also include a

vector of potential confounder variables (e.g., age, gender, education, and/or other socioeconomic indicators) to account for possible imbalances between study arms. All analyses will be based on the intention-to-treat principle whereby all clients will be included in the analysis irrespective of whether they complied with the intervention in the outlet at which they sought care (e.g. even if they did not use the ACT subsidy if they tested positive in an outlet in Arms 2 and 3 that received the client-directed intervention).

Given that the literature indicates that when there are fewer than 40 clusters in a cRCT, small sample correction methods should be used to ensure that standard error estimates are correctly estimated when using GEE to analyze binary outcomes, and given that the size of the cRCTs in each country are close to this cut-off, we plan to adopt the use of the Kauerman-Carroll correction to avoid any possible problems [34, 35]. We will compare secondary outcomes using the same modeling approach.

7 SUBJECT CONFIDENTIALITY

Participant confidentiality will be maintained during and after the interview in several ways:

- The interview will be conducted in a private place where the interview cannot be overheard.
- Interviewers will be trained in research integrity and ethics, including protecting participant information.
- Informed consent will be conducted verbally, thereby reducing the risk of a breach of client confidentiality by eliminating any paper record of clients' participation in the study with signature. Only those enrolling in Quality Assurance subsample will be asked to provide written consent. Consent forms will be stored in a locked cabinet to which only the site coordinator has access. No study identifiers will be recorded on the consent form ensuring that participant data cannot be linked to the names on the consent document.
- Data will be collected on tablets which will be encrypted and password protected.
- Individuals will be assigned a unique study ID. No information that could be used to identify the participant will be recorded such as names, identification numbers, dates of birth or address.
- Anyone older than 80 years of age will be recorded as '80.'
- Data will be transferred from the tablet to a secure, password protected computer once per week.
- Data will be stored on an encrypted, password protected computer and backed up on Duke Box. The data will only be accessible to the Data Manager and the PI. It will be reviewed regularly to ensure quality and completeness.
- Only fully de-identified data will be provided to other study personnel or statisticians. Only fully de-identified data will be shared.

7.1 Future Use of Stored Specimens

Not applicable.

8 INFORMED CONSENT PROCESS

Pharmacies will be approached and the study described. If they agree to participate, they will be asked to consent to a baseline survey that collects details about the outlet (Appendix 4,5). This will be conducted in a private area and at a time that is convenient for their business.

Informed consent will be obtained prior to collecting any participant information for research purposes. Clients will be approached as they leave the pharmacy and asked if they would be willing to participate in a brief interview. If so, the interviewer will conduct screening and consent in a private area away from other clients. The client will be given time to ask questions and will be asked to give verbal consent for the interview, which will be documented by the interviewer. The interviewer will explain the purpose of the study, the risks, benefits, and safeguards in place to protect the participant’s information. A printed copy of the consent script will be available for them to read and to keep. Contact information for the Study Coordinator or PI will be provided so the participant can ask questions after the interview.

No individual identifying information will be collected during the interview, therefore withdrawing consent after the interview is closed will not be possible. To mitigate this, the interviewer will confirm consent by asking at the end of the survey if the participant is comfortable with all the answers and agrees for their information to be retained.

Only those enrolling in Quality Assurance subsample will be asked to provide written consent for RDT conducted by the study team. Consent forms will be stored in a locked cabinet to which only the site coordinator has access. No study identifiers will be recorded on the consent form ensuring that participant data cannot be linked to the names on the consent document.

Focus group participants and interviewees will be asked to provide written consent for participation in qualitative studies.

8.1 Informed Consent/Assent Process (in Case of a Minor or Others Unable to Consent for Themselves)

The study will only enroll minor participants present with a parent or guardian and all questions will be directed to the parent/guardian. Therefore, we do not anticipate interacting with minors or needing assent from the minor.

We will not enroll individuals who are unable to consent for themselves such as those who are mentally impaired or are experiencing impaired consciousness.

We will only enroll adults seeking treatment for themselves for the Quality Assurance Exit Testing subsample. We will not enroll children in the exit testing.

9 RESULT DISSEMINATION PLAN

The results from this trial will be disseminated to the county and sub-county health teams, National Malaria Control Program, and the Director of Medical Services through meetings and a policy brief.

We shall also support the sub-county health teams to disseminate the results at the community level through barazas or CHWs depending on plans for scale-up coming from national partners.

10 LITERATURE REFERENCES

1. Gething, P.W., et al., *Mapping Plasmodium falciparum Mortality in Africa between 1990 and 2015.* N Engl J Med, 2016. **375**(25): p. 2435-2445.
2. World Health Organization., *World Malaria Report 2016*. 2016, World Health Organization: Geneva.
3. Unitaid., *Global Malaria Diagnostic and Artemesinin Treatment Commodities in Demand Forecast 2017-2020*. 2018, World Health Organization: Vernier, Switzerland.
4. Lindblade, K.A., et al., *Mortality of sick children after outpatient treatment at first-level health facilities in rural western Kenya.* Trop Med Int Health, 2007. **12**(10): p. 1258-68.
5. Opoka, R.O., et al., *Inpatient mortality in children with clinically diagnosed malaria as compared with microscopically confirmed malaria.* Pediatr Infect Dis J, 2008. **27**(4): p. 319-24.
6. Nsanzabana, C., et al., *Quantifying the evolution and impact of antimalarial drug resistance: drug use, spread of resistance, and drug failure over a 12-year period in Papua New Guinea.* J Infect Dis, 2010. **201**(3): p. 435-43.
7. Hastings, I.M., *Modelling parasite drug resistance: lessons for management and control strategies.* Trop Med Int Health, 2001. **6**(11): p. 883-90.
8. O'Meara, W.P., D.L. Smith, and F.E. McKenzie, *Potential impact of intermittent preventive treatment (IPT) on spread of drug-resistant malaria.* PLoS Med, 2006. **3**(5): p. e141.
9. Cohen, J.M., et al., *Public health. Optimizing investments in malaria treatment and diagnosis.* Science, 2012. **338**(6107): p. 612-4.
10. World Health Organization., *World Malaria Report 2015*. 2015, World Health Organization: Geneva.
11. Mbonye, A.K., et al., *Treatment of fevers prior to introducing rapid diagnostic tests for malaria in registered drug shops in Uganda.* Malar J, 2013. **12**: p. 131.
12. Briggs, M.A., et al., *Prevalence of malaria parasitemia and purchase of artemisinin-based combination therapies (ACTs) among drug shop clients in two regions in Tanzania with ACT subsidies.* PLoS One, 2014. **9**(4): p. e94074.
13. Nwokolo, E., et al., *Misuse of Artemisinin Combination Therapies by Clients of Medicine Retailers Suspected to Have Malaria Without Prior Parasitological Confirmation in Nigeria.* Int J Health Policy Manag, 2018. **7**(6): p. 542-548.
14. Montagu, D., et al., *Recent trends in working with the private sector to improve basic healthcare: a review of evidence and interventions.* Health Policy Plan, 2016. **31**(8): p. 1117-32.
15. Sudhinaraset, M., et al., *What is the role of informal healthcare providers in developing countries? A systematic review.* PLoS One, 2013. **8**(2): p. e54978.
16. Boyce, M.R. and W.P. O'Meara, *Use of malaria RDTs in various health contexts across sub-Saharan Africa: a systematic review.* BMC Public Health, 2017. **17**(1): p. 470.
17. Hanson, K. and C. Goodman, *Testing times: trends in availability, price, and market share of malaria diagnostics in the public and private healthcare sector across eight sub-Saharan African countries from 2009 to 2015.* Malar J, 2017. **16**(1): p. 205.
18. Cohen, J., et al., *Introducing rapid diagnostic tests for malaria to drug shops in Uganda: a cluster-randomized controlled trial.* Bulletin of the World Health Organization, 2015. **93**(3): p. 142-151.

19. Maloney, K., et al., *Expanding access to parasite-based malaria diagnosis through retail drug shops in Tanzania: evidence from a randomized trial and implications for treatment.* Malar J, 2017. **16**(1): p. 6.
20. Onwujekwe, O., et al., *Effectiveness of Provider and Community Interventions to Improve Treatment of Uncomplicated Malaria in Nigeria: A Cluster Randomized Controlled Trial.* PLoS One, 2015. **10**(8): p. e0133832.
21. Awor, P., et al., *Increased access to care and appropriateness of treatment at private sector drug shops with integrated management of malaria, pneumonia and diarrhoea: a quasi-experimental study in Uganda.* PLoS One, 2014. **9**(12): p. e115440.
22. Mbonye, A.K., et al., *A Cluster Randomised Trial Introducing Rapid Diagnostic Tests into Registered Drug Shops in Uganda: Impact on Appropriate Treatment of Malaria.* PLoS One, 2015. **10**(7): p. e0129545.
23. Ansah, E.K., et al., *The impact of providing rapid diagnostic malaria tests on fever management in the private retail sector in Ghana: a cluster randomized trial.* Bmj, 2015. **350**: p. h1019.
24. Visser, T., et al., *Introducing malaria rapid diagnostic tests in private medicine retail outlets: A systematic literature review.* PLoS One, 2017. **12**(3): p. e0173093.
25. Hansen, K.S., T.H. Lesner, and L.P. Osterdal, *Optimal price subsidies for appropriate malaria testing and treatment behaviour.* Malar J, 2016. **15**(1): p. 534.
26. Hutchinson, E., et al., *Introducing rapid tests for malaria into the retail sector: what are the unintended consequences?* BMJ Glob Health, 2017. **2**(1): p. e000067.
27. Hayes, R.J. and L.H. Moulton, *Cluster Randomized Trials*, ed. C.H.C.P.I. Statistics. 2009.
28. Aickin, M. and H. Gensler, *Adjusting for multiple testing when reporting research results: the Bonferroni vs Holm methods.* Am J Public Health, 1996. **86**(5): p. 726-8.
29. Prudhomme O'Meara, W., et al., *Assessing the independent and combined effects of subsidies for antimalarials and rapid diagnostic testing on fever management decisions in the retail sector: results from a factorial randomised trial in western Kenya.* BMJ Glob Health, 2016. **1**(2): p. e000101.
30. Zou, G., *A modified poisson regression approach to prospective studies with binary data.* Am J Epidemiol, 2004. **159**(7): p. 702-6.
31. Zou, G.Y. and A. Donner, *Extension of the modified Poisson regression model to prospective studies with correlated binary data.* Stat Methods Med Res, 2013. **22**(6): p. 661-70.
32. Zeger, S.L., K.Y. Liang, and P.S. Albert, *Models for longitudinal data: a generalized estimating equation approach.* Biometrics, 1988. **44**(4): p. 1049-60.
33. Preisser, J.S., et al., *An integrated population-averaged approach to the design, analysis and sample size determination of cluster-unit trials.* Stat Med, 2003. **22**(8): p. 1235-54.
34. Adeyi, O. and R. Atun, *Innovating for impact: the Affordable Medicines Facility-malaria (AMFm).* Nat Med, 2009. **15**(9): p. 991.
35. Amfm Independent Evaluation Team, et al., *Independent Evaluation of Phase 1 of the Affordable Medicines Facility - malaria (AMFm), Multi-Country Independent Evaluation Final Report*. 2012, ICF International and London School of Hygiene and Tropical Medicine: Calverton, Maryland and London.

SUPPLEMENTS/APPENDICES

Appendix A.1

Aim 2: TESTsmART Participant exit interview

SCREENING QUESTIONS

Number	QUESTION	RESPONSE	SKIP
**0.01**	**Did the person meet all of the inclusion criteria?**	1. Yes<br>2. No	If yes, skip to 0.03
**0.02**	**If no, why were they excluded from participating in the study today? (select all that apply)**	1. Person did not have a fever or malaria-like illness<br>2. Patient with malaria-like symptoms not present<br>3. Patient younger than 1 year of age<br>4. Had symptoms of severe malaria and referred for care<br>5. Took antimalarial in the last seven days for current illness<br>6. No parent or guardian present	STOP
**0.03**	**Did the person consent to participate in the study?**	1. Yes<br>2. No	If No, STOP

GENERAL STUDY INFORMATION

Number	QUESTION	RESPONSE	SKIP
**0.1**	**Date**	MM/YYYY	
**0.2**	**Participant ID**		
**0.3**	**Outlet ID**		
**0.4**	**Interviewer ID**		
**0.5**	**Language of interview**	1. English<br>2. Swahili<br>3. Bukusu<br>4. Other	

SECTION 1: RESPONDENT INFORMATION

Number	QUESTION	RESPONSE	SKIP
**1.1**	**Who is the respondent?**	Adult with fever………………………1<br>Guardian of the child…………………2	→Q1.5
**1.2**	**What is your relationship with the child?**	Parent………………………………..1<br>Grandmother/grandfather…………….2<br>Brother/Sister………………………….3<br>Uncle/Aunt…………………………..…4<br>Other…………………………………5<br>Specify:	
**1.3**	**Gender of the child**	Female………………………...1<br>Male…………………………...0	
**1.4**	**How old is the child?**	__ ___ . __ years	
**1.5**	**Gender of the respondent**	Female………………………...1<br>Male…………………………...0	
**1.6**	**How old are you?**<br>*[In the case the child is ill, please collect this information for the parent/guardian of the child]*	_____ _____ years	

SECTION 2: CURRENT ILLNESS

Number	QUESTION	RESPONSE	SKIP
**2.1**	**Which symptoms do you/your child have or had in the last 24 hours?**<br>*[Mark all that apply]*	1. Fever<br>2. Nausea<br>3. Headache<br>4. Body aches<br>5. Vomiting<br>6. Shivering<br>7. Stomach ache<br>8. Other______________________<br>99. Don't know	

**2.2**	**How serious is this illness in your opinion?**<br><br>*[Guardian of the child can guess how the child feels]*	1. Not very serious/minor<br>2. Moderate<br>3. Very serious	
**2.3**	**How many days ago did the symptoms start?**	_______days	99=Don't know
**2.4**	**How likely is that the illness that you/your child have today is malaria?**	1. Not possible<br>2. Unlikely but not impossible<br>3. 50/50<br>4. Likely<br>5. Absolutely sure<br><br>99. Don't know	
**2.5**	**Have you sought treatment or care elsewhere for this illness?**	1. Yes<br>2. No<br><br>99. Don't know	If no or don't know, go to 3.1
**2.6**	**What did you do?** *Check all that apply*	1. Visit hospital<br>2. Visit government health center/dispensary<br>3. Visit private clinic<br>4. Visit private laboratory<br>5. Visit pharmacy/chemist<br>6. Buy medicine at general shop<br>7. Gave medicine available at home<br>8. Visit traditional healers<br>9. Visit religious/cultural healers<br>10. Visit CHW<br>11. Other______________________<br><br>99. Don't know (i.e., another caregiver was involved)	
**2.7**	**Why did you come to this outlet?**	1. Medicine was not available in other facilities<br>2. RDT tests were not available in other facilities<br>3. It's the closest chemist shop to me<br>4. This chemist shop has better prices<br>5. I was referred here<br>6. OTHER<br>99. Don't know (i.e., another caregiver was involved)	

**2.8**	**What kind of malaria test did you have from another provider/facility/lab before coming here today?**	1. Microscopy<br>2. RDT<br>3. Don't know<br>4. No malaria test	If 4, go to 3.1
**2.9**	**Malaria test results from elsewhere**	1. Negative – report observed<br>2. Positive – report observed<br>3. Reported negative by respondent<br>4. Report positive by respondent<br>5. Doesn't recall result	
**2.10**	**Do you have a copy of the test result?**	1. No, nothing written<br>2. Yes	
**2.11**	**How likely is it that the result of that test was correct?**<br>***Only if tested elsewhere before coming to the shop today***	1. Not possible<br>2. Unlikely but not impossible<br>3. 50/50<br>4. Likely<br>5. Absolutely sure<br>Don't know	
**2.12**	**Have you taken/given to the child any medication for this illness since it started?**	1. Yes<br>2. No<br>99. Don't know	If 1, go to 2.12<br>If 2 or 99, go to Section 3
**2.13**	**If so, which medicines (select all that apply)**	1. AL (Lonart/CoArtem/Artefan)<br>2. Other ACT (DHAP, DP, Duocotexin, P-alaxin)<br>3. Monotherapy (Artesunate, Quinine, Chloroquine, SP/Fansidar)<br>4. Antibiotic (Amoxyl/Ceptrin, Metronidazole/Flagyl/Ampicillin)<br>5. Painkiller/fever medecine (Panadol/Brufen/Hedex/ Action/Maramoja)<br>6. Cough medicine or decongestant<br>7. Other________________<br>99. Don't know	If 1, 2 or 3 STOP
**2.14**	**Did you come to the outlet today with a prescription from another provider/facility/lab?**	1. Yes<br>2. No	If 2 skip to Section 3.
**2.15**	**If yes, what was the prescription for?**	1. AL (Lonart/CoArtem/Artefan)<br>2. Other ACT (DHAP, DP, Duocotexin, P-alaxin)	

		3. Monotherapy (Artesunate, Quinine, Chloroquine, SP/Fansidar)<br>4. Antibiotic (Amoxyl/Ceptrin, Metronidazole/Flagyl/Ampicillin)<br>5. Painkiller/fever medecine (Panadol/Brufen/Hedex/ Action/Maramoja)<br>6. Cough medicine or decongestant<br>7. Other_________________<br>99. Don't know	
**2.16**	**Did the client show documentation of the prescription?**	1. No<br>2. Yes, a written prescription from the provider/facility/lab<br>3. Yes, other (Specify:___________)	

Section 3: Test at Outlet

Number	QUESTION	RESPONSE	SKIP
**3.1**	**Did you (or your child) have your blood tested for malaria today at the outlet?**	1. Yes<br>2. No	If yes skip to 3.3
**3.2**	**Why not? (Mark all that apply)**	1. Too expensive<br>2. RDT was not offered<br>3. RDT not in stock<br>4. No time<br>5. Already sure illness is malaria<br>6. Already sure illness is not malaria<br>7. Don't want to get finger pricked<br>8. Was tested elsewhere before coming<br>9. Had a prescription for drug I needed before coming<br>99. Other<br>Specify: _________________	Next skip to section 4
**3.3**	**RDT results from outlet (self-report)**	1. Negative<br>2. Positive<br>3. Invalid	
**3.4**	**How much did you pay for the RDT?**	__________ KES	

**3.5**	**How likely is it that the result of the test was correct?** ***Only if tested***	1. Not possible<br>2. Unlikely but not impossible<br>3. 50/50<br>4. Likely<br>5. Absolutely sure<br>Don't know	

Section 4: Purchase Questions

Number	QUESTION	RESPONSE	SKIP
**4.1**	**Which medicine(s) did you obtain from the outlet today to treat your/your child's illness**<br>**Choose all that apply,**	1. AL (Lonart/CoArtem/Artefan)<br>2. Other ACT (DHAP, DP, Duocotexin, P-alaxin)<br>3. Other antimalarial (Artesunate injection, Quinine, Chloroquine, SP/Fansidar)<br>4. Antibiotic (Amoxyl/Ceptrin, Metronidazole/Flagyl/Ampicillin)<br>5. Painkiller/fever medicine (Panadol/Brufen/Hedex/ Action/Maramoja)<br>6. Cough medicine or decongestant<br>7. Other________________<br>8. None<br>99. Don't know	If 2, 3, 4, 5, 6, 7 or 99 Skip to 4.4<br><br>If 8, skip to 5.1
**4.2**	**How much did you pay today for the ACT?**<br><br>**(The amount refers to how much <u>money</u> respondent paid for the ACT. Don't add the amount of the voucher if any was used-the amount should be between 0-5000, or 9999 for don't know)**	ACT= ________KES<br><br>OR<br><br>Don't know/remember…9999	
**4.3**	**How much did you spend at the outlet today? (*Total cost to client including RDT, ACT and any other drugs*)**	_________ KES	
**4.4**	**Was there any discount for your ACT at the outlet today?**	1. Yes<br>2. No<br>3. Don't know	

**4.5**	**I would like to record information about each drug you purchased today. May I see the packaging?**	1. Type ________________<br>2. Brand Name ________________<br>3. Price ________________ KES<br>4. *Did participant show you the drug?*<br>1. Yes<br>2. No	Repeat for every drug purchased for illness

Section 5: Household Characteristics

[In the case the child is ill, please collect this information for the parent/guardian of the child.]

**Number**	**QUESTION**	**RESPONSE**	**SKIP**
**5.1**	**What is the main source of drinking water for your household?**	1. Piped water/Public Tap/borehole<br>2. Unprotected well<br>3. Protected well<br>4. Protected Spring<br>5. Unprotected Spring<br>6. Surface water (river, dam, lake, pond, stream, canal, irrigation channel)<br>7. Rain water<br>8. Bottled water<br>9. Other..	
**5.2**	**Does your household have the following items:**		
**a)**	**Electricity?**	1. Yes<br>2. No<br>99. Don’t know	
**b)**	**A television?**	1. Yes<br>2. No<br>99. Don’t know	
**c)**	**A refrigerator?**	1. Yes<br>2. No<br>99. Don’t know	
**d)**	**A radio?**	1. Yes	

		2. No<br><br>99. Don't know	
**e)**	**A mobile phone (at least one member of the household has)?**	1. Yes<br>2. No<br><br>99. Don't know	
**f)**	**A motorcycle (at least one member of the household has)?**	1. Yes<br>2. No<br><br>99. Don't know	
**g)**	**A car/truck?**	1. Yes<br>2. No<br><br>99. Don't know	
**h)**	**A bank account (at least one member of the household has)?**	1. Yes<br>2. No<br><br>99. Don't know	
**5.3**	**How many of the following livestock does your household have?**	1. None<br><br>99. Don't know	
**a)**	**Cows**		
**b)**	**Sheep**		
**c)**	**Goats**		
**d)**	**Pigs**		
**5.4**	**What kind of toilet does your household have?**	2. Flush or pour flush toilet<br>3. VIP / Ventilated improved pit latrine<br>4. Pit latrine with slab<br>5. Pit latrine without slab<br>6. Composting toilet<br>7. Bucket toilet<br>8. No facility / bush / field<br>9. Other (Please specify):<br>____________________	
**5.5**	**What type of fuel does your household <u>mainly</u> use for cooking?**	1. Liquefied petroleum gas<br>2. Paraffin/Kerosene<br>3. Charcoal<br>4. Firewood<br>5. Dung	

		6. Biogas<br>7. Crop residue<br>8. Other (Specify)______________ _	
**5.6**	**Do you/your family own the house you live in?**	1. Own the house<br>2. Rent the house	
**5.7**	**What is the <u>main</u> material of the floor in your house?**	3. Earthen<br>4. Cement<br>5. Floor Tiles<br>6. Wood planks<br>7. Polished wood<br>8. Other (please specify) ________________________	
**5.8**	**What is the <u>main</u> material of the walls in your house?**	1. Stone<br>2. Brick<br>3. Timber<br>4. Iron Sheet<br>5. Mud<br>6. Wood<br>7. Cement<br>8. Other (please specify)______________	
**5.9**	**What is the <u>main</u> material of the roof of your house?**	1. Iron sheets<br>2. Roof tiles<br>3. Grass Thatched<br>4. Wood<br>5. Other (please specify)_______________	
**5.10**	**How many acres/hectares/feet of land for farming does your household own?**	1.None .... 0<br>2. Acres______<br>3. Square Feet (xx by xx)_______<br>9999. Don't know	
**5.11**	**What is the highest level of schooling you completed?**	1. None<br>2. Primary<br>3. Secondary<br>4. College<br>5. University	
**5.12**	**What is your primary occupation?**	Agriculture<br>1. Farming/Livestock keeping	

		Paid employee<br>2. Government or parastatal<br>3. Private(specify: ....)<br>4. Self-employed With employees<br>5. Without employees (e.g. motorcycle taxi, vendor)<br>6. Unpaid family helper in a business<br><br>Other (Not in agriculture)<br>7. Casual worker/day laborer<br>8. House help<br>9. Homemaker<br>10. Student<br>11. Not available to work<br>12. Other (Specify) _____________	
